# A Functional Model of Sensemaking in a Neurocognitive Architecture

**DOI:** 10.1155/2013/921695

**Published:** 2013-11-05

**Authors:** Christian Lebiere, Peter Pirolli, Robert Thomson, Jaehyon Paik, Matthew Rutledge-Taylor, James Staszewski, John R. Anderson

**Affiliations:** ^1^Carnegie Mellon University, Pittsburgh, PA 15213, USA; ^2^Palo Alto Research Center, Palo Alto, CA 94304, USA

## Abstract

Sensemaking is the active process of constructing a meaningful representation (i.e., making sense) of some complex aspect of the world. In relation to intelligence analysis, sensemaking is the act of finding and interpreting relevant facts amongst the sea of incoming reports, images, and intelligence. We present a cognitive model of core information-foraging and hypothesis-updating sensemaking processes applied to complex spatial probability estimation and decision-making tasks. While the model was developed in a hybrid symbolic-statistical cognitive architecture, its correspondence to neural frameworks in terms of both structure and mechanisms provided a direct bridge between rational and neural levels of description. Compared against data from two participant groups, the model correctly predicted both the presence and degree of four biases: confirmation, anchoring and adjustment, representativeness, and probability matching. It also favorably predicted human performance in generating probability distributions across categories, assigning resources based on these distributions, and selecting relevant features given a prior probability distribution. This model provides a constrained theoretical framework describing cognitive biases as arising from three interacting factors: the structure of the task environment, the mechanisms and limitations of the cognitive architecture, and the use of strategies to adapt to the dual constraints of cognition and the environment.

## 1. Introduction

We present a computational cognitive model, developed in the ACT-R architecture [1, 2], of several core information-foraging and hypothesis-updating processes involved in a complex sensemaking task. Sensemaking [3–6] is a concept that has been used to define a class of activities and tasks in which there is an active seeking and processing of information to achieve understanding about some state of affairs in the world. Complex tasks in intelligence analysis and situation awareness have frequently been cited as examples of sensemaking [3–5]. Sensemaking, as in *to make sense*, implies an active process to construct a meaningful and functional representation of some aspects of the world. A variety of theories and perspectives on sensemaking have been developed in psychology [3, 4], human-computer interaction [6], information and library science [7], and in organizational science [8]. In this paper we present a cognitive model of basic sensemaking processes for an intelligence analysis task.

A major concern in the intelligence community is the impact of cognitive biases on the accuracy of analyses [9]. Two prominent biases are confirmation bias, in which an analyst disproportionately considers information that supports the current hypothesis, and anchoring bias, in which an initial judgment is insufficiently revised in the face of new evidence. In the task used in this paper, sensemaking is instantiated in terms of estimation of probability distributions over hypothesis space. Rational Bayesian optima are defined over those distributions, with cognitive biases defined as deviations from those optima. In this framework, confirmation bias can then be defined as a distribution “peakier” than the Bayesian optimum, whereas anchoring bias is a flatter-than-rational distribution reflecting an insufficient adjustment from the original uniform prior. We present simulation results that exhibit several cognitive biases, including confirmation bias, anchoring and adjustment, probability matching, and base-rate neglect. Those biases are not engineered in the model but rather result from the interaction of the structure and statistics of the task, the structure and mechanisms of our cognitive architecture, and the strategies that we select to perform the former using the latter.

**Figure 1:**
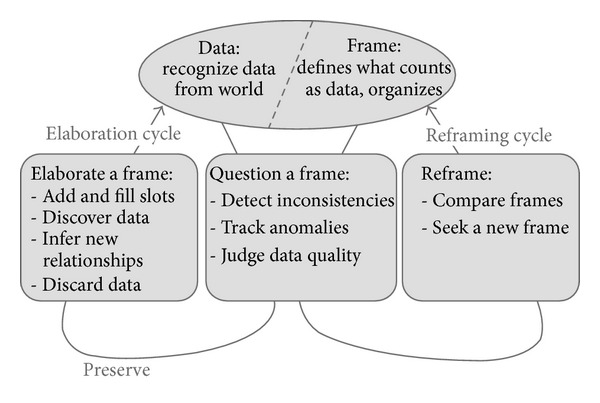
The Data-Frame model of sensemaking. Image reproduced by Klein et al. [4].

Figure 1 presents the Data/Frame theory of sensemaking [3]. The Data/Frame theory assumes that meaningful mental representations called *frames* define what counts as *data* and how those data are structured for mental processing [4]. A similar conceptual model was employed in Pirolli and Card [5] to perform a cognitive task analysis of intelligence analysis [10–12]. Frames can be expressed in a variety of forms including stories, maps, organizational diagrams, or scripts. Whereas frames define and shape data, new data can evoke changes to frames. In this framework, sensemaking can involve elaboration of a frame (e.g., filling in details), questioning a frame (e.g., due to the detection of anomalies), or reframing (e.g., rejecting a frame and replacing it with another). The Data/Frame theory proposes that backward-looking processes are involved in forming mental models that explain past events, and forward-looking mental simulations are involved in predicting how future events will unfold. We describe how frames can be represented in a cognitive architecture and how the architectural mechanisms can implement general sensemaking processes. We then demonstrate how the dynamics of sensemaking processes in a cognitive architecture can give rise to cognitive biases in an emergent way.

The structure of this paper is as follows.  Section 2 defines the AHA (Abducting Hotspots of Activity) experiment consisting of a suite of six sensemaking tasks of increasing complexity.  Section 3 outlines our cognitive modeling approach to sensemaking: it describes the ACT-R architecture, how it is used to prototype neural models, which cognitive functions compose the model, and how four cognitive biases can be accounted for by the model.  Section 4 presents the measures used to assess the cognitive biases in the AHA framework and then compares human and model results.  Section 5 presents a test of the model's generalization on a data set that was unavailable at the time of initial model development. Finally, Section 6 summarizes our account of cognitive biases centered around the mechanisms and limitations of cognitive architectures, the heuristic that these mechanisms use to adapt to the structure of the task, and their interaction with the task environment.

## 2. The Task Environment

The AHA experiment consists of a series of six tasks developed as part of the IARPA (Intelligence Advanced Research Projects Activity), ICArUS (Integrated Cognitive-neuroscience Architectures for the Understanding of Sensemaking) program, whose goal is to drive the development of integrated neurocognitive models of heuristic and biases in decision-making in the context of intelligence analysis. The AHA tasks can be subdivided into two classes: the first focusing on learning the statistical patterns of events located on a map-like layout and generating probability distributions of category membership based on the spatial location and frequency of these events (Tasks 1–3) and the second requiring the application of probabilistic decision rules about different features displayed on similar map-like layouts in order to generate and revise probability distributions of category membership (Tasks 4–6).

The AHA tasks simulate the analysis of artificial geospatial data presented in a manner consistent with and informed by current intelligence doctrine (Geospatial Intelligence Basic Doctrine; http://www.fas.org/irp/agency/nga/doctrine.pdf). The tasks involve the presentation of multiple features consistent with intelligence data, which are presented in a GIS (Geographic Information System) display not unlike Google maps (https://maps.google.com). These features include

 HUMINT: information collected by human sources such as detecting the location of events,
 IMINT: information collected from imagery of buildings, roads, and terrain elements,
 MOVINT: analysis of moving objects such as traffic density,
 SIGINT: analysis of signals and communications,
 SOCINT: analysis of social customs and attitudes of people, communities, and culture.

**Figure 2:**
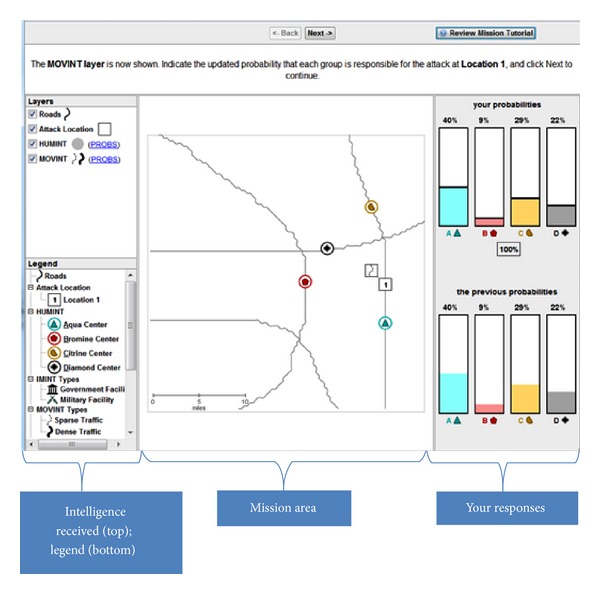
The image is a sample of the display in Task 4. To the left is a legend explaining all the symbols on the map (center). To the right are the probability distributions for the four event categories (both for the current and prior layer of information). The panel across the top provides step-by-step instructions for participants.

The display (see Figure 2) includes access to the mission tutorial and instructions (the top-right corner), a legend to understand the symbols on the map (the left pane), the map (the center pane), and participants' current and past responses (the right pane).

For Tasks 1–3, the flow of an average trial proceeds according to the following general outline. First, participants perceive a series of events (SIGACTs; SIGnals of ACTivity) labeled according to which category the event belonged. Categories were both color- and shape-coded, with the appropriate label {Aqua, Bromine, Citrine, or Diamond} listed in the legend. After perceiving the series of events, a probe event is displayed (represented as a “?” on the display). Participants were asked to generate a center of activity (e.g., prototype) for each category's events, reflect on how strongly they believed the probe belonged to each category, and generate a probability estimate for each category (summed to 100% across all groups) using the sliders or by incrementing the counters presented on the right side of the Task interface. As an aid, the interface automatically normalized the total probability such that the total probability summed across each category equaled 100%. Participants were not provided feedback at this step. Scoring was determined by comparing participants distributions to an optimal Bayesian solution (see Section 4 for a detailed description of how the probability estimate scores are calculated). Using these scores it was possible to determine certain biases. For instance, participants' probability estimates that exhibited lower entropy than an optimal Bayes model would be considered to exhibit a confirmation bias, while probability estimates having higher entropy than an optimal Bayes model would be considered to exhibit an anchoring bias.

After finalizing their probability estimates, participants were then asked to allocate resources (using the same right-side interface as probability estimates) to each category with the goal of maximizing their resource allocation score, which was the amount of resources allocated to the correct category. Participants would receive feedback only on their resource allocation score. For Tasks 1–3, the resource allocation response was a forced-choice decision to allocate 100% of their resources to a single category. If that category produced the probe event, then the resource allocation score was 100 out of 100 for choosing the correct category, otherwise 0 out of 100 for choosing an incorrect category. Following this feedback, the next trial commenced.

For Tasks 4–6, the flow of an average trial was structurally different as intelligence “features,” governed by probabilistic decision rules (see Table 1), were presented sequentially as separate layers of information on the display. These Tasks required reasoning based on rules concerning the relation of observed evidence to the likelihood of an unknown event belonging to each of four different categories. Participants updated their beliefs (i.e., likelihoods) after each layer of information (i.e., feature) was presented, based on the probabilistic decision rules described in Table 1.

For instance, in Task 4, after determining the center of activity for each category (similar in mechanism to Tasks 1–3) and reporting an initial probability estimate, the SOCINT (SOCial INTelligence) layer would be presented by displaying color-coded regions on the display representing each category's boundary. After reviewing the information presented by the SOCINT layer, participants were required to update their likelihoods based on this information and the corresponding probabilistic decision rule.

When all the layers have been presented (two layers in Task 4, five layers in Task 5, and four layers in Task 6), participants were required to generate a resource allocation. In these Tasks, the resource allocation response was produced using the same interface as probability estimates. For instance, assuming that resources were allocated such that {A = 40%, B = 30%, C = 20%, D = 10%} and if the probe belonged to category A (i.e., that A was the “ground truth”), then the participant would receive a score of 40 out of 100, whereas if the probe instead belonged to category B, they would score 30 points. The resource allocation score provided the means of measuring the probability matching bias. The optimal solution (assuming one could correctly predict the right category with over 25% accuracy) would be to always allocate 100% of one's resources to the category with the highest probability. Allocating anything less than that could be considered an instance of probability matching.

Finally, participants were not allowed to use any assistive device (e.g., pen, paper, calculator, or other external devices), as the intent of the Task was to measure how well participants were able to make rapid probability estimates without any external aids.

### 2.1. Task 1

**Figure 3:**
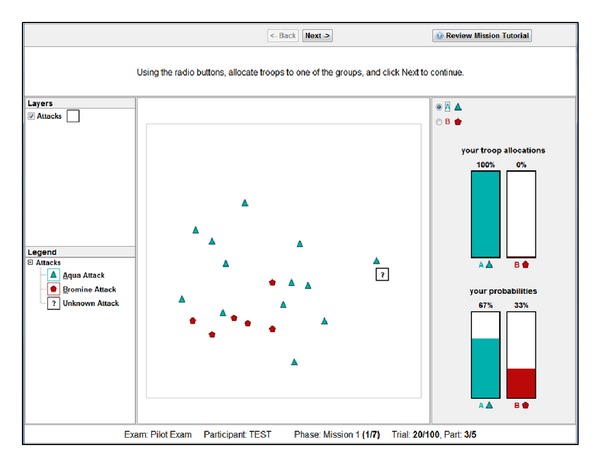
Sample output from Task 1. Participants must generate the likelihood that a probe event (denoted by the “?”) was produced by each category and then perform a forced-choice resource allocation to maximize their trial score. Likelihoods are based on the distance from each category's centroid and the frequency of events. For instance, Aqua has a higher likelihood because its centroid is closer to the probe and it has a higher frequency (i.e., more events) than Bromine.

In Task 1, participants predicted the likelihood that a probe event belonged to either of two categories {Aqua or Bromine}. Categories were defined by a dispersion value around a centroid location (e.g., central tendency), with individual events produced probabilistically by sampling in a Gaussian window using a similar function as seen in prototype distortion methodologies from dot pattern categorization studies [13]. The interface was presented spatially on a computer screen (see Figure 3) in a 100 × 100 grid pattern (representing 30 square miles; grid not shown).

Participants were instructed to learn about each category's tendencies according to three features: the category's center of activity (i.e., centroid), the dispersion associated with each category's events, and the frequency of events for each category. Using these three features, participants determined the likelihood that the probe event belonged to each category.

A trial consisted of 10 events, with 9 events presented sequentially at various locations about the interface, with participants required to click “next” after perceiving each event. The 10th event was the probe event, which was presented as a “?” on the interface. Each participant completed 10 trials, with events accumulating across trials such that 100 events were present on the interface by the end of the task.

After perceiving the probe event, participants were instructed to generate likelihoods that the probe event belonged to each category based on all the events that they have seen not just the recent events from the current trial. These likelihoods were expressed on a scale from 1 to 99% for each category and summing to 100% across both categories. If necessary, the interface would automatically normalize all likelihoods into probabilities summing to 100%.

Finally, participants entered a forced-choice resource allocation response, analogous to a measure of certainty. Resource allocation was a forced-choice decision to allocate 100% of their resources to a single category. If that category produced the probe event, then the participant would receive feedback that was either 100 out of 100 for choosing the correct category or 0 out of 100 for choosing an incorrect category. Following this feedback, the next trial commenced.

### 2.2. Task 2

**Figure 4:**
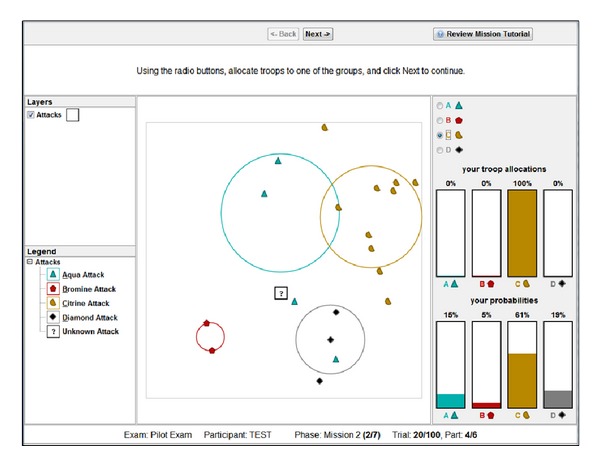
Sample output from Task 2. Participants must generate the likelihood that a probe event (denoted by the “?”) was produced by each category and then do a forced-choice resource allocation to maximize their trial score. In addition, participants had to draw a 2-to-1 boundary for each category whose boundary encapsulates 2/3 of that category's events and whose center represents the center of activity for that category. Likelihoods are based on the distance from each category's centroid and the frequency of events. For instance, Citrine has the highest likelihood because it has a higher frequency than the other categories, while Diamond has a marginally higher likelihood than Aqua and Bromine because it has the closest distance.

In Task 2, participants predicted the likelihood that a probe event belonged to either of four categories {Aqua, Bromine, Citrine, or Diamond}. The interface and procedure were similar to Task 1, with the following differences. A trial consisted of 20 events, with 19 events presented sequentially at various locations about the interface. The 20th event was the probe event, which was presented as a “?” on the interface. Each participant completed 5 trials, with events accumulating across trials such that 100 events were present on the interface by the end of the task. Participants were further required to estimate each category's centroid and dispersion by drawing a circle for each category representing a 2-to-1 boundary with 2/3 of the category's events inside the circle and 1/3 outside (see Figure 4). Participants clicked with the mouse to set the centroid and dragged out with the mouse to capture the 2-to-1 boundary, releasing the mouse to set the position. It was possible to adjust both the position and dispersion for each category after their initial set. Estimating category centroids and dispersion preceded generating likelihoods.

Finally, participants entered a similar forced-choice resource allocation response as in Task 1. Resource allocation was a forced-choice decision to allocate 100% of their resources to a single category. If that category produced the probe event, then the participant would receive feedback that was either 100 out of 100 for choosing the correct category or 0 of out 100 for choosing an incorrect category. Following this feedback, the next trial commenced.

### 2.3. Task 3

In Task 3, participants predicted the likelihood that a probe event belonged to either of four categories similar to Task 2, with the following differences. Instead of the interface instantiating a blank grid, it displayed a network of roads. Events were only placed along roads, and participants were instructed to estimate distance along roads rather than “as the crow flies.” In addition, participants no longer had to draw the 2-to-1 boundaries but instead only identify the location of the category centroid.

**Figure 5:**
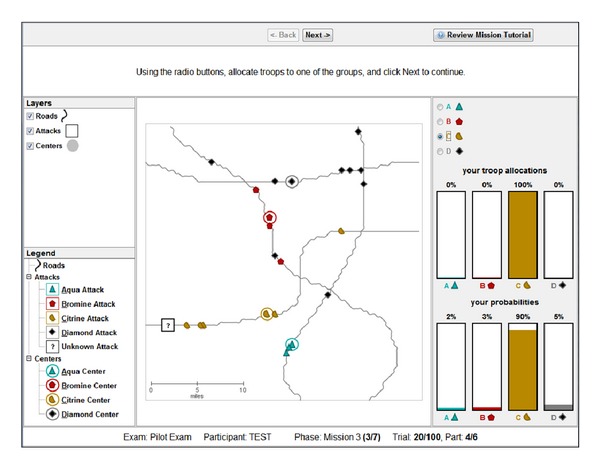
Sample output from Task 3. Participants must generate the likelihood that a probe event (denoted by the “?”) was produced by each category and then do a forced-choice resource allocation to maximize their trial score. Likelihoods are based on the road distance from each category's centroid and the frequency of events. For instance, Citrine has the highest likelihood because it is the closest category.

A trial consisted of 20 events, with 19 events presented sequentially at various locations about the interface. The 20th event was the probe event, which was presented as a “?” on the interface. Each participant completed 5 trials, with events accumulating across trials such that 100 events were present on the interface by the end of the task. Participants were further required to estimate each category's centroid by placing a circle for each category (see Figure 5). Participants clicked with the mouse to set the centroid. It was possible to adjust the position for each category after the initial set. Based on the requirement to judge road distance, Task 3 (and Task 4) involved additional visual problem solving strategies (e.g., spatial path planning and curve tracing) [14].

Finally, participants entered a similar forced-choice resource allocation response as in Task 2. Resource allocation was a forced-choice decision to allocate 100% of their resources to a single category. If that category produced the probe event, then the participant would receive feedback that was either 100 out of 100 for choosing the correct category or 0 out of 100 for choosing an incorrect category. Following this feedback, the next trial commenced.

### 2.4. Task 4

**Figure 6:**
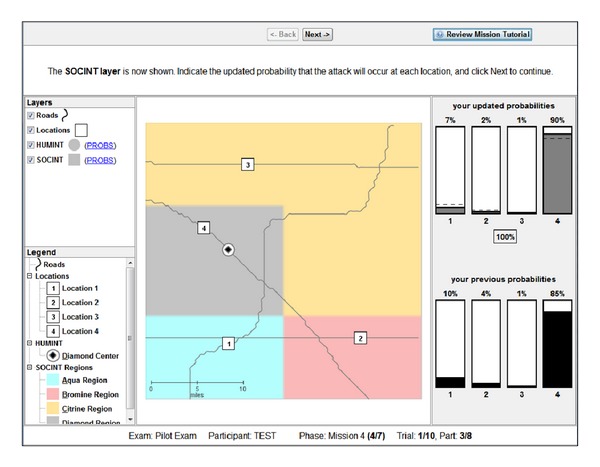
Sample output from Task 4. Participants must generate the likelihood that a probe event (denoted by the Diamond) was produced by each category (1–4), first by the HUMINT layer (distance from category centroids to probe event) and then by the SOCINT layer (likelihoods are doubled for the category in whose region the probe event falls). Finally, participants allocate resources to maximize their trial score. For instance, category 4 has the highest likelihood because it is the closest category and the probe falls within its boundary.

Beginning with Task 4, instead of gathering information from a sequence of events, participants instead generated and updated likelihoods after being presented with a number of features as separate layers of information. These features were governed by probabilistic decision rules [15] described previously in Table 1. In Task 4, two features were presented to participants in a fixed order. The first layer was HUMINT (HUMan INTelligence), which revealed the location of the category centroid for each category. The second layer was SOCINT (SOCial INTelligence), which revealed color-coded regions on the display representing each category's boundary (see Figure 6). If a probe event occurred in a given category's boundary, then the probability that the probe belonged to that category was twice as high as the event belonging to any of the other categories.

Participants were instructed that the feature layers provided “clues” revealing intelligence data (called INTs) and the probabilistic decisions rules (called PROBs rules) provided means to interpret INTs. Participants were instructed to refer to the PROBs handbook (based on Table 1; see the appendix for the complete handbook), which was accessible by clicking on the particular layer in the legend on the left side of the display or by reviewing the mission tutorial in the top-right corner. They were further instructed that each feature layer was independent of other layers.

The same simulated geospatial display from Task 3 was used in Task 4; however, instead of a trial consisting of a series of individual events, a trial instead consisted of reasoning from category centroids to a probe event by updating likelihoods after each new feature was revealed. A trial consisted of two features presented in sequence (HUMINT and SOCINT, resp.). The HUMINT layer revealed the centroids for each category along with the probe event. Participants reported likelihoods for each category {1, 2, 3, or  4} based on the road distance between the probe and each category's centroid. Similar to previous tasks, likelihoods were automatically normalized to a probability distribution (i.e., summing to 100%). After this initial distribution was input, the SOCINT feature was presented by breaking the display down into four colored regions representing probabilistic category boundaries. Using these boundaries, participants applied the SOCINT rule and updated their probability distribution.

Once their revised probability distribution was entered, participants were required to generate a resource allocation. The resource allocation response was produced using the same interface as probability estimates. For instance, assuming that resources were allocated such that {1 = 40%, 2 = 30%, 3 = 20%, 4 = 10%} and if the probe belonged to category 1 (i.e., that 1 was the “ground truth”), then the participant would receive a score of 40 out of 100, whereas if the probe instead belonged to category 2, they would score 30 points. After completing their resource allocation, the display was reset and a new trial started.

Participants completed 10 trials. Unlike Tasks 1–3, each trial was presented on a unique road network with all four category locations presented in a unique location. 

### 2.5. Task 5

**Figure 7:**
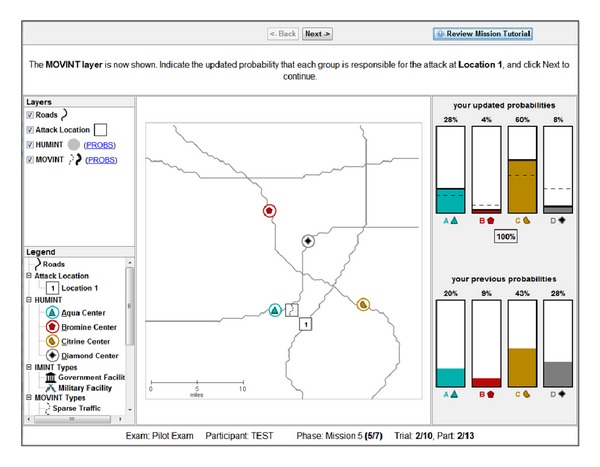
Sample output from Task 5. Participants must generate the likelihood that a probe event (denoted by the probe event “1”) was produced by each category. The HUMINT layer is always displayed first, and the initial probability distribution based on road distance is provided to participants. Participants must update this initial distribution as new features are revealed. In the current example, the likelihoods of categories A and C are increased due to the MOVINT layer revealing sparse traffic at the probe event location (see PROBs rules in Table 1).

In Task 5, all five features were revealed to participants in each trial, with the HUMINT feature always revealed first (and the rest presented in a random order). Thus, participants began each trial with each category's centroid presented on the interface and the Bayesian optimal probability distribution already input on the right-side response panel (see Figure 7). The goal of Task 5 was to examine how participants fused multiple layers of information together. Unlike Task 4, the correct probability distribution for HUMINT was provided to participants. This was done both to reduce the variance in initial probabilities (due to the noisiness of spatial road distance judgments) and also to reduce participant fatigue. After perceiving HUMINT and being provided the correct probability distribution, each of the four remaining features (SOCINT, IMINT, MOVINT, and SIGINT on a single category) was revealed in a random order. After each feature was revealed, participants updated their probability distribution based on applying the corresponding decision rules. Similar to Task 4, after the final feature was revealed, participants allocated resources.

The same methodology was used as for Task 4, only with five layers of features presented instead of two. Participants reported likelihoods for each category {Aqua, Bromine, Citrine, or Diamond} based on the information revealed by the feature at each layer according to the rules of the PROBs handbook. Likelihoods were automatically normalized to a probability distribution (i.e., summing to 100%). After HUMINT was revealed, four other features (SOCINT, MOVINT, IMINT, and SIGINT) were revealed in random order. The SOCINT feature was presented by breaking the display down into four colored regions representing probabilistic category boundaries. The IMINT (IMagery INTelligence) feature was presented by showing either a government or military building at the probe location. The MOVINT (MOVement INTelligence) feature was presented by showing either sparse or dense traffic at the probe location. Finally, the SIGINT (SIGnal INTelligence) feature was presented by showing either the presence or absence of chatter for a specific category at the probe location. After each feature was revealed, participants applied the relevant PROBs rule and updated their probability distribution.

After all feature layers were revealed and probability distributions were revised, participants were required to generate a resource allocation. The resource allocation response was produced using the same interface as in Task 4. After completing their resource allocation, the display was reset and a new trial started. Participants completed 10 trials. Note that participants needed to update likelihoods four times per trial (thus 40 times in total) in addition to a single resource allocation per trial (10 total). Similar to Task 4, each trial was presented on a unique road network with all four category locations presented in a unique location.

### 2.6. Task 6

**Figure 8:**
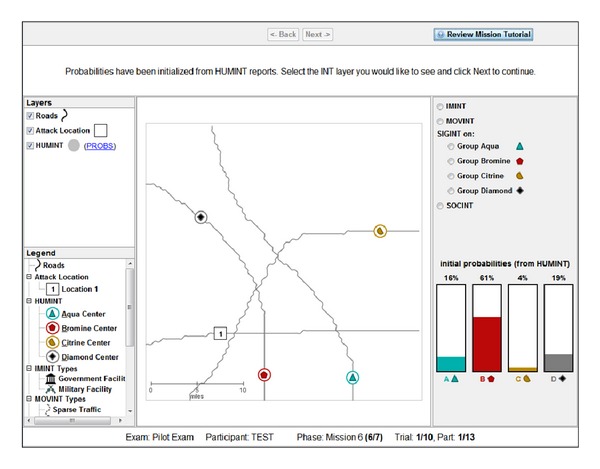
Sample output from Task 6. Participants must generate the likelihood that a probe event (denoted by the probe event “1”) was produced by each category. The HUMINT layer is always displayed first, and the initial probability distribution based on road distance is provided to participants. Participants must update this initial distribution as new features are revealed. In the current example, the likelihoods of categories A and C are increased due to the MOVINT layer revealing sparse traffic at the probe event location.

In Task 6, participants were able to choose three of four possible features to be revealed, in addition to the order in which they are revealed (see Figure 8). The goal of Task 6 was to determine participants' choices and ordering in selecting features (which we refer to as layer selection). This methodology determined whether participants were biased to pick features whose corresponding decision rule confirmed their leading hypothesis or possibly maximized potential information gain. Participants were instructed to choose layers that maximized information at each step to increase the likelihood of a single category being responsible for the event.

As for Task 5, a trial began by perceiving the HUMINT layer and being provided the correct probability distribution. Participants must then choose a feature to be revealed (SOCINT, IMINT, MOVINT, or SIGINT on a single category). When participants chose the SIGINT layer, they needed to further specify which category they were inspecting (listening for chatter). After the chosen feature was revealed, participants updated their probability distribution based on applying the corresponding decision rules. This process was repeated twice more with different features, for a total of three layers being chosen. Participants must update category likelihoods {Aqua, Bromine, Citrine, or Diamond} after each layer was revealed based on the information provided by the corresponding feature at each layer according to the rules of the PROBs handbook. As in the other tasks, likelihoods were automatically normalized to sum to 100% across categories. Note that with only three layer selection choices, participants were not able to reveal one feature on each trial.

After participants completed the process of choosing a feature and updating their likelihoods for each of three iterations, participants were required to generate a resource allocation. The resource allocation response was produced using the same interface as in Tasks 4-5. After completing their resource allocation, the display was reset and a new trial commenced. Participants completed 10 trials. Note that, with three layer selections, participants actually updated probabilities 30 times (3 times per trial), in addition to allocating resources once for each trial. Similar to Tasks 4-5, each trial was presented on a unique road network with all four category locations presented in a unique location.

## 3. An ACT-R Model of Sensemaking

### 3.1. Overview of ACT-R

Our aim has been to develop a functional model of several core information-foraging and hypothesis-updating processes involved in sensemaking. We do this by developing ACT-R models to specify how elementary cognitive modules and processes are marshaled to produce observed sensemaking behavior in a set of complex geospatial intelligence Tasks. These tasks involve an iterative process of obtaining new evidence from available sources and using that evidence to update hypotheses about potential outcomes. One purpose of the ACT-R functional model is to provide a roadmap of the interaction and sequencing of neural modules in producing task performance (see next section). A second purpose is to identify and understand a core set mechanisms for producing cognitive biases observed in the selection and weighting of evidence in information foraging (e.g., confirmation bias).

**Figure 9:**
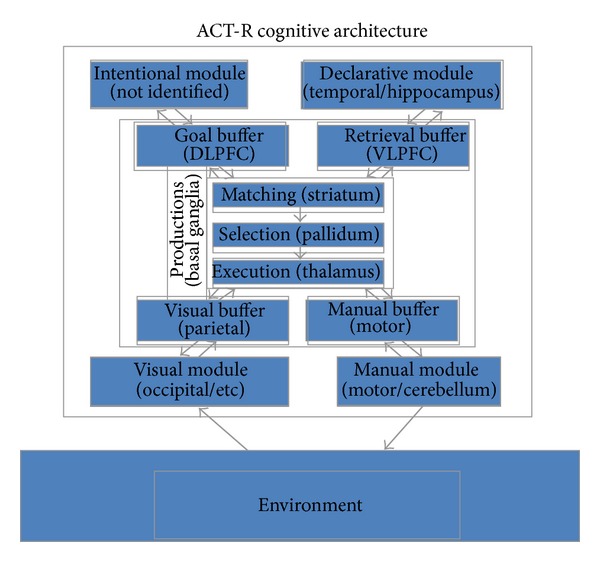
ACT-R functions as a production system architecture with multiple modules corresponding to different kinds of perception, action, and cognitive information stores. Modules have been identified with specific brain regions. In addition to those shown above, the Imaginal module has been associated with posterior parietal activation.

The ACT-R architecture (see Figure 9) is organized as a set of modules, each devoted to processing a particular kind of information, which are integrated and coordinated through a centralized production system module. Each module is assumed to access and deposit information into buffers associated with the module, and the central production system can only respond to the contents of the buffers not the internal encapsulated processing of the modules. Each module, including the production module, has been correlated with activation in particular brain locations [1]. For instance, the visual module (occipital cortex and others) and visual buffers (parietal cortex) keep track of objects and locations in the visual field. The manual module (motor cortex; cerebellum) and manual buffer (motor cortex) are associated with control of the hands. The declarative module (temporal lobe; hippocampus) and retrieval buffer (ventrolateral prefrontal cortex) are associated with the retrieval and awareness of information from long-term declarative memory. The goal buffer (dorsolateral prefrontal cortex) keeps track of the goals and internal state of the system in problem solving. Finally, the production system (basal ganglia) is associated with matching the contents of module buffers and coordinating their activity. The production includes components for pattern matching (striatum), conflict resolution (pallidum), and execution (thalamus). A production rule can be thought of as a formal specification of the flow of information from buffered information in the cortex to the basal ganglia and back again [16].

The declarative memory module and production system module, respectively, store and retrieve information that corresponds to declarative knowledge and procedural knowledge [17]. Declarative knowledge is the kind of knowledge that a person can attend to, reflect upon, and usually articulate in some way (e.g., by declaring it verbally or by gesture). Procedural knowledge consists of the skills we display in our behavior, generally without conscious awareness. Declarative knowledge in ACT-R is represented formally in terms of chunks [18, 19]. The information in the declarative memory module corresponds to personal episodic and semantic knowledge that promotes long-term coherence in behavior. In this sense a chunk is like a data frame, integrating information available in a common context at a particular point in time in a single representational structure. The goal module stores and retrieves information that represents the internal intention and problem solving state of the system and provides local coherence to behavior.

Chunks are retrieved from long-term declarative memory by an activation process (see Table 2 for a list of retrieval mechanisms in ACT-R). Each chunk has a base-level activation that reflects its recency and frequency of occurrence. Activation spreads from the current focus of attention, including goals, through associations among chunks in declarative memory. These associations are built up from experience, and they reflect how chunks cooccur in cognitive processing. The spread of activation from one cognitive structure to another is determined by weighting values on the associations among chunks. These weights determine the rate of activation flow among chunks. Chunks are compared to the desired retrieval pattern using a partial matching mechanism that subtracts from the activation of a chunk its degree of mismatch to the desired pattern, additively for each component of the pattern and corresponding chunk value. Finally, noise is added to chunk activations to make retrieval a probabilistic process governed by a Boltzmann (softmax) distribution. While the most active chunk is usually retrieved, a blending process [20] can also be applied which returns a derived output reflecting the similarity between the values of the content of all chunks, weighted by their retrieval probabilities reflecting their activations and partial-matching scores. This blending process will be used intensively in the model since it provides both a tractable way to learn to perform decisions in continuous domains such as the probability spaces of the AHA framework and a direct abstraction to the storage and retrieval of information in neural models (see next section).

Production rules are used to represent procedural knowledge in ACT-R. That is, they specify procedures that represent and apply cognitive skill (know-how) in the current context and how to retrieve and modify information in the buffers and transfer it to other modules. In ACT-R, each production rule has conditions that specify structures that are matched in buffers corresponding to information from the external world or other internal modules. Each production rule has actions that specify changes to be made to the buffers.

ACT-R uses a mix of parallel and serial processing. Modules may process information in parallel with one another. So, for instance, the visual modules and the motor modules may both operate at the same time. However, there are two serial bottlenecks in process. First, only one production may be executed during a cycle. Second, each module is limited to placing a single chunk in a buffer. In general, multiple production rules can be applied at any point. Production utilities, learned using a reinforcement learning scheme, are used to select the single rule that fires. As for declarative memory retrieval, production selection is a probabilistic process.

Cognitive model development in ACT-R [21] is in part derived from the rational analysis of the task and information structures in the external environment (e.g., the design of the tasks being simulated or the structure of a graphical user interface), the constraints of the ACT-R architecture, and guidelines from previous models of similar tasks. A successful design pattern in specifying cognitive process sequencing in ACT-R [21] is to decompose a complex task to the level of unit tasks [22]. Card et al. [22] suggested that unit tasks control immediate behavior. Unit tasks empirically take about 10 seconds. To an approximation, unit tasks are where “the rubber of rationality meets the mechanistic road.” To an approximation, the structure of behavior above the unit task level largely reflects a rational structuring of the task within the constraints of the environment, whereas the structure within and below the unit task level reflects cognitive and biological mechanisms, in accordance with Newell's bands of cognition [23]. Accordingly, in ACT-R, unit tasks are implemented by specific goal types that control productions that represent the cognitive skills for solving those tasks.

ACT-R has been the basis for several decades of research on learning complex cognitive tasks such as algebra and programming [24, 25]. In general, the long-run outcome of learning such tasks is a large set of highly situation-specific productions whose application is sharply tuned by ACT-R utility mechanisms (a form of reinforcement learning). However, it is also generally assumed that achieving such expert levels of learning requires 1000s of hours of experience. We assume that the participants in the AHA tasks will not have the opportunity to achieve such levels of expertise. Instead, we hypothesize that participants will rely on direct recognition or recall of relevant experience from declarative memory to guide their thinking or, failing that, will heuristically interpret and deliberate through the rules and evidence provided in the challenge tasks. This compute-versus-retrieve process is another design pattern that typically structures ACT-R models [21]. The notion that learners have a general-purpose mechanism whereby situation-action-outcome observations are stored and retrieved as chunks in ACT-R declarative memory is derived from instance-based learning theory (IBLT) [26, 27]. Gonzalez et al. [26] present arguments that IBLT is particularly pertinent to modeling naturalistic decision making in complex dynamic situations, and many of those arguments would transfer to making the case that IBLT is appropriate for sensemaking.

Relevant to the Bayesian inspiration for the AHA tasks, ACT-R's subsymbolic activation formula approximates Bayesian inference by framing activation as log-likelihoods, base-level activation (*B*_*i*_) as the prior, the sum of spreading activation and partial matching as the likelihood adjustment factor(s), and the final chunk activation (*A*_*i*_) as the posterior. The retrieved chunk has an activation that satisfies the maximum likelihood equation. ACT-R provides constraint to the Bayesian framework through the activation equation and production system. The calculation of base levels (i.e., priors) occurs within both neurally and behaviorally consistent equations (see Table 2) providing for behaviorally relevant memory effects like recency and frequency while also providing a constrained mechanism for obtaining priors (i.e., driven by experience).

In addition, the limitations on matching in the production system provide constraints to the Bayesian hypothesis space and, as a result, the kinds of inferences that can be made. For instance, there are constraints on the kinds of matching that can be accomplished (e.g., no disjunction, matching only to specific chunk types within buffers), and, while user-specified productions can be task-constrained, the production system can generate novel productions (through proceduralization of declarative knowledge) using production compilation. In addition, the choice of which production to fire (conflict resolution) also constrains which chunks (i.e., hypotheses) will be recalled (limiting the hypothesis space) and are also subject to learning via production utilities.

It has been argued that ACT-R's numerous parameters do not provide sufficient constraint on modeling endeavors. However, the use of community and research-justified default values, the practice of removing parameters by developing more automatized mechanisms, and the development of common modeling paradigms—such as instance-based learning theory—mitigate these criticisms by limiting degrees of freedom in the architecture and thus constraining the kinds of models that can be developed and encouraging their integration.

### 3.2. ACT-R Prototyping for Neural Models

ACT-R can be used in a prototyping role for neural models such as Emergent, which uses the Leabra learning rule [28]. In ACT-R, models can be quickly developed and tested, and the results of these models then help inform modeling efforts and direct training strategies in Emergent models [29, 30]. ACT-R models can be created quickly because ACT-R models accept predominantly functional specifications, yet they produce neurally relevant results. The ACT-R architecture is also flexible enough that innovations made in neurocomputational models can be implemented (to a degree of abstraction) within new ACT-R modules [31].

There are several points of contact between ACT-R and Emergent, the most tangible of which is a commitment to neural localization of architectural constructs in both architectures (see Figure 9). In both architectures a central control module located in the basal ganglia collects inputs from a variety of cortical areas and outputs primarily to the frontal cortex, which maintains task relevant information [16, 32]. Additionally, both include a dedicated declarative/episodic memory system in the hippocampus and associated cortical structures. Lastly, both account for sensory and motor processing in the posterior cortex.

The architectures differ in that the brain regions are explicitly modeled in Emergent, whereas they are implicit in ACT-R. In ACT-R the basal ganglia are associated with the production system; the frontal cortex with the goal module; the parietal cortex with the imaginal module; the hippocampus with the declarative memory module; and finally the posterior cortices with the manual, vocal, aural, and vision modules. This compatibility of ACT-R and Emergent has been realized elsewhere by the development of SAL (Synthesis of ACT-R and Leabra/Emergent), a hybrid architecture that combines ACT-R and Emergent and exploits the relative strengths of each [33]. Thus, ACT-R connects to the underlying neural theory of Emergent and can provide meaningful guidance to the development of neural models of complex tasks, such as sensemaking.

In effect, ACT-R models provide a high-level specification of the information flows that will take place in the neural model between component regions implemented in Emergent. Since ACT-R models have been targeted at precisely this level of description, they can provide for just the right level of abstraction while ignoring many implementational details (e.g., number of connections) at the neural level. Conceptually, the ACT-R architecture provides a bridge between the rational Bayesian level and the detailed neural level. In terms of Marr [34] levels of analysis, the Bayesian characterization of the task solutions is situated at the computational level, describing the computations that should be performed without specifying how. An ACT-R account of the tasks is at the algorithmic/representational level, specifying what representations are created, which mechanisms are used to manipulate them, and which structure constrains both representations and processes. Finally, a neural account is situated at the physical/implementational level, fully specifying all the details of how the computations are to be carried out in the brain. Thus, just as in Marr's analysis it would not make sense to try to bridge directly the highest and lowest levels; a functional cognitive architecture such as ACT-R provides a critical link between abstract computational specifications such as Bayesian rational norms and highly detailed neural mechanisms and representations.

Moreover, ACT-R does not just provide any intermediate level of abstraction between computational and implementational levels in a broad modular sense. Rather, just as the ACT-R mechanisms have formal Bayesian underpinnings, they also have a direct correspondence to neural mechanisms and representations. The fundamental characteristics of modern neural modeling frameworks are distributed representations, local learning rules, and training of networks from sets of input-output instances [35].

Distributed representations are captured in ACT-R through similarities between chunks (and other sets of values such as number magnitudes) that can be thought of as corresponding to the dotproduct between distributed representations of the corresponding chunks. The generalization process operates over distributed representations in neural networks, effectively matching the learned weights from the input units resulting in a unit containing the representation of the current input. This is implemented in ACT-R using a partial matching mechanism that combines a chunk's activation during the memory retrieval process with its degree of match to the requested pattern as determined by the similarities between chunk contents and pattern [36].

Local learning rules in neural networks are used to adjust weights between units based on information flowing through the network. The base-level and associative learning mechanisms in ACT-R perform a similar function in the same manner. Both have Bayesian underpinnings [37] but also direct correspondence to neural mechanisms. Base-level learning is used to adjust the activation of a chunk based on its history of use, especially its frequency and recency of access. This corresponds to learning the bias of a unit in a network, determining its initial activation which is added to inputs from other units. Associative learning adjusts the strengths of association between chunks to reflect their degree of coactivation. While the original formulation was Bayesian in nature, a new characterization makes the link to Hebbian-like learning explicit, in particular introducing the same positive-negative learning phases as found in many connectionist learning algorithms including Leabra [31].

Neural models are created by a combination of modeler-designed structure and training that adjusts the network's weights in response to external inputs. The instance-based learning approach in ACT-R similarly combines a representational structure provided by the modeler with content acquired from experience in the form of chunks that represent individual problem instances. The set of chunks stored in declarative memory as a result can be thought of as the equivalent to the set of training instances given to the neural network. While the network compiles those instances into weights during training, ACT-R can instead dynamically blend those chunks together during memory retrieval to produce an aggregate response that reflects the consensus of all chunks, weighted by their probability of retrieval reflecting the activation processes described above [20].

Thus, ACT-R models can be used to prototype neural models because they share both a common structure of information flow as well as a direct correspondence from the more abstract (hence tractable) representations and mechanisms at the symbolic/subsymbolic level and those at the neural level.

### 3.3. Cognitive Functions Engaged in the AHA Tasks

The integrated ACT-R model of the AHA tasks has been used to prototype many cognitive effects in neural models including generating category prototypes of centroids from SIGACT events (centroid generation) [29], spatial path planning along road networks [30], adjusting probabilities based on incoming information [29], choosing how many resources to allocate given a set of probabilities and prior experience [29], and selecting additional intelligence layers (see Table 3 for an overview). The model's output compared favorably with human behavioral data and provides a comprehensive explanation for the origins of cognitive biases in the AHA framework, most prominently the anchoring and adjustment bias. All the functions described below were integrated in a single ACT-R model that performed all 6 AHA tasks using the same parameters. That model was learned across trials and tasks. We will describe later in details how its performance in the later trials of a task can depend critically upon its experience in the earlier trials (even just the first trial), in particular leading to a distinct conservatism bias. Similarly, its performance in the later tasks depends upon its experience in earlier tasks, leading directly to probability matching bias in resource allocation.

The model performs the task in the same manner as human subjects. Instructions such as the probabilistic decision rules are represented in declarative memory for later retrieval when needed. The model perceives the events, represents them in the imaginal buffer, and then stores them in declarative memory where they influence future judgments. In Tasks 1–3, the model uses those past events in memory to generate the category centroid when given a probe. In Tasks 3-4, the model programmatically parses the map to represent the road network declaratively and then uses that declarative representation to generate paths and estimate road distances. In all tasks, probability adjustment is performed using the same instance-based mechanism, with experience from earlier tasks accumulated in memory for use in later tasks. Resource allocation is also performed in all tasks using the same instance-based approach, with results from forced-choice selections in Tasks 1–3 fundamentally affecting choices in later Tasks 4–6. Finally, layer selection in Task 6 uses experiences in Tasks 4-5 to generate estimates of information gain and select the most promising layer. Thus the integrated model brings to bear constraints from all tasks and functions.

#### 3.3.1. Centroid Generation

The ACT-R integrated model generates category centroids (i.e., the prototype or central tendency of the events) in Tasks 1–3 by aggregating overall of the representations of events (e.g., spatial-context frames) in memory via the blended memory retrieval mechanism. The goal buffer maintains task-relevant top-down information while the blending buffer creates/updates centroids from both the set of perceived SIGACTs to date and prior created centroids. Centroid generation approximates a stochastic least-MSE derived from distance and based on the 2D Cartesian coordinates of the individual SIGACTs. Specifically, the mismatch penalty (*P*_*i*_) used in the blended retrieval is a linear difference:

(1) $$\begin{array}{c} P_{i}=\frac{2\cdot \left|d_{1}-d_{2}\right|}{*\max\_\text{range}*}, \end{array}$$

where *d* is the perceived distance and ∗max_range∗ is the size of the display (100 units). The imaginal buffer (correlated with parietal activity) is used to hold blended chunks before being committed to declarative memory. When centroids are generated directly from SIGACTs, the blending process reflects a disproportionate influence of the most recent events given their higher base-level activation. A strategy to combat this recency bias consisted of generating a final response by performing a blended retrieval over the current and past centroids, thereby giving more weight to earlier SIGACTs. This is because the influence of earlier centroids has been compounded over the subsequent blended retrievals, essentially factoring earlier SIGACTs into more centroids. This second-order blended retrieval is done for each category across their prior existing centroids, which we refer to as the generation of a centroid-of-centroids. This blending over centroids effectively implements an anchoring-and-adjustment process where each new centroid estimate is a combination of the previous ones together with the new evidence. A fundamental difference with traditional implementation of anchoring-and-adjustment heuristic is that this process is entirely constrained by the architectural mechanisms (especially blending) and does not involve additional degrees of freedom. Moreover, because there are an equal number of centroid chunks (one per category created after each trial), there is no effect of category base rate on the model's later probability judgments, even though the base rate for each category is implicitly available in the model based on the number of recallable events.

#### 3.3.2. Path Planning

The ACT-R model uses the declarative memory and visual modules to implement path planning, which simulate many of the parietal functionalities that were later implemented in a Leabra model [30]. Two examples include

1. perceptually segmenting the road network so that the model only attends to task-relevant perceptual elements,
2. implementing visual curve tracing to model the psychophysics of how humans estimate curved roads.

The model segments the road network in Tasks 3-4 into smaller elements and then focuses perceptual processes such as curve tracing, distance estimation, and path planning on these smaller road segments [38]. Specifically, the model identifies the intersections of different roads as highly salient HUMINT features and then splits the road network into road segments consisting of two intersections (as the ends of the segment), the general direction of the road and the length of road. Intersections are generally represented as a particular location on the display in Cartesian *X*-*Y* coordinates.

For each trial, the probe location is also represented as a local HUMINT feature, and, in Task 3, the individual events are represented as local HUMINT features for the purposes of calculating category centroids. At the end of each trial, the probe is functionally removed from the path planning model, although a memory trace of the previous location still remains in declarative memory.

We have implemented a multistrategy hill-climber to perform path planning. The model starts with a category centroid and appends contiguous road segments until the probe location is reached (i.e., the model is generating and updating a spatial-context frame). The path planning “decision-making” is a function of ACT-R's partial matching. In partial matching, a similarity is computed between a source object and all target objects that fit a set of matching criteria. This similarity score is weighted by the mismatch penalty scaling factor. The hill-climber matches across multiple values such as segment length and remaining distance to probe location. In general, for all road segments adjoining the currently retrieved intersection or category centroid, the model will tend to pick the segment where the next intersection is nearest to the probe. This is repeated until the segment with the probe location is retrieved. These strategies are not necessarily explicit but are instead meant to simulate the cognitive weights of different perceptual factors (e.g., distance, direction, and length) guiding attentional processes. The partial matching function generates probabilities from distances and calculates similarity between distances using the same mismatch penalty as in Tasks 1 and 2.

Human performance data on mental curve tracing [14] show that participants take longer to mentally trace along a sharper curve than a relatively narrower curve. This relation is roughly linear (with increased underestimation of total curve length at farther curves) and holds along the range of visual sizes of roads that were seen in the AHA tasks. This modeling assumption is drawn from the large body of the literature on visuospatial representation and mental scanning [39]. When road network is parsed, a perceived length is assigned to each road segment. This length is more or less represented veridically in declarative memory. The dissociation between a veridical perceived magnitude of distance and a postprocessed cognitive distance estimate is consistent with prior literature [40]. We represent a cognitive distance estimate using Stevens' Power Law [41]. Stevens' Power Law is a well-studied relationship between the magnitude of a stimulus and its perceived intensity and serves as a simple yet powerful abstraction of many low-level visual processes not currently modeled in ACT-R.

The function uses the ratio of “as the cow walks” distance to “as the crow flies” distance to create an estimate of curve complexity [41]. The higher the curve complexity, the curvier the road. To represent the relative underestimation of distance for curvier segments, this ratio is raised to an exponent of .82 [41–43]. The effect of this parameter is that, for each unit increase in veridical distance, the perceived distance is increased by a lesser extent. The closer the exponent to 1, the more veridical the perception, and the closer to zero, the more the distance will be underestimated. This value for curve complexity is then multiplied by a factor representing straight-line distance estimation performance (1.02) [43–45]:

(2) $$\begin{array}{c} {D=\left\{1.02\left(\frac{{\text{Cow}}_{\text{Walk}}}{{\text{Crow}}_{\text{Flies}}}\right)+{\text{Crow}}_{\text{Flies}}\;\right\}}^{.82}, \end{array}$$

where *D* is the cognitive judgment of distance for the road segment, Cow_Walk_ is the veridical perception of the curvature of the road, and Crow_Flies_ is the veridical perception of the Euclidean distance between the source and target locations. The factor of 1.02 represents a slight overestimation of smaller straight-line distances. Similar to the exponent, any factor above unity represents an overestimation of distance and any factor below unity represents an underestimation of distance.

#### 3.3.3. Probability Adjustment

Lebiere [20] proposed a model of cognitive arithmetic that used blended retrieval of arithmetic facts to generate estimates of answers without explicit computations. The model was driven by number of similarities that correspond to distributed representations for number magnitudes in the neural model and more generally to our sense of numbers [46]. It used partial matching to match facts related to the problem and blended retrievals to merge them together and derive an aggregate estimated answer. The model reproduced a number of characteristics of the distribution of errors in elementary school children, including both table and nontable errors, error gradients around the correct answer, higher correct percentage for tie problems, and, most relevant here, a skew toward underestimating answers, a bias consistent with anchoring and adjustment processes.

**Figure 10:**
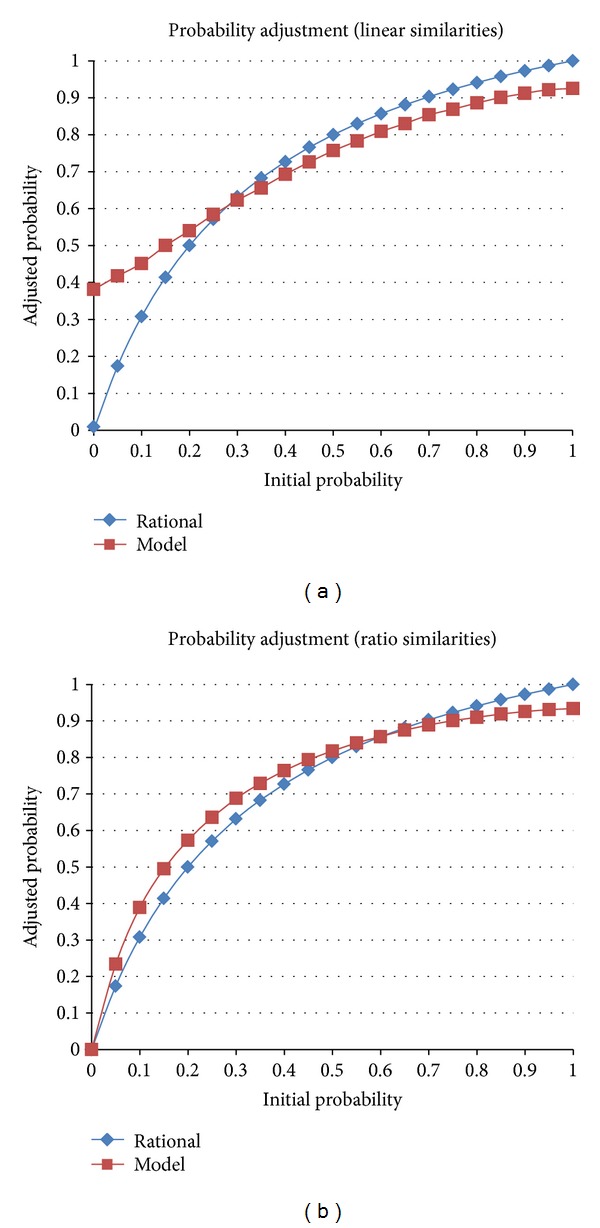
Results from an ACT-R model of probability adjustment with linear (a) and ratio (b) similarities.

To leverage this approach for probability adjustment, the ACT-R model's memory was populated with a range of facts consisting of triplets: an initial probability, an adjustment factor, and the resulting probability. These triplets form the building blocks of the implementation of instance-based learning theory [47] and correspond roughly to the notion of a decision frame [3, 4]. In the AHA framework, the factor is set by the explicit rules of the task (e.g., an event in a category boundary is twice as likely to belong to that category). The model is then seeded with a set of chunks that correspond to a range of initial probabilities and an adjustment factor together with the posterior probability that would result from multiplying the initial probability by the adjustment factor, then normalizing it. When the model is asked to estimate the resulting probability for a given prior and multiplying factor, it simply performs a blended retrieval specifying prior and factor and outputs the posterior probability that represents the blended consensus of the seeded chunks.  Figure 10 displays systematic results of this process, averaged over a thousand runs, given the variations in answers resulting from activation noise in the retrieval process. When provided with linear similarities between probabilities (and factors), the primary effect is an underestimation of the adjusted probability for much of the initial probability range, with an overestimate on the lower end of the range, especially for initial values close to 0. The latter effect is largely a result of the linear form of the number similarities function. While linear similarities are simple, they fail to scale both to values near zero and to large values.

A better estimate of similarities in neural representations of numbers is a ratio function, as reflected in single cell recordings [1]. This increases dissimilarities of the numbers near zero and scales up to arbitrarily large numbers. When using a ratio similarity function, the effects from the linear similarity function are preserved, but the substantial overestimate for the lower end of the probability range is considerably reduced. While the magnitude of the biases can be modulated somewhat by architectural parameters such as the mismatch penalty (scaling the similarities) or the activation noise (controlling the stochasticity of memory retrieval), the effects themselves are a priori predictions of the architecture, in particular its theoretical constraints on memory retrieval.

Particular to the integrated model of the AHA tasks, the mismatch penalty (*P*_*i*_) was computed as a linear difference:

(3) $$\begin{array}{c} P_{i}=2*\left|M_{k}-M_{j}\right|, \end{array}$$

where *M*_*k*_ is the possible target probability and *M*_*j*_ is the probability in the blended retrieval specification. As will be described below, this linear difference matches extremely well to human behavioral data.

The results of the ACT-R mechanism for probability adjustment provided a benchmark against which neural models were evaluated and were used to generate training instances for the neural model which had already embodied the proper bias. In our opinion, this constitutes a novel way to use functional models to quickly prototype and interpret neural models.

#### 3.3.4. Resource Allocation

The resource allocation mechanism in the model makes use of the same instance-based learning paradigm as the probability adjustment mechanism. This unified mechanism has no explicit strategies but instead learns to allocate resources according to the outcomes of past decisions. The model generates a resource allocation distribution by focusing on the leading category and determining how many resources to allocate to that category. The remaining resources are divided amongst the remaining three categories in proportion to their assigned probabilities. This instance-based learning not only occurs during Tasks 4–6 but also in Tasks 1–3 for forced-choice allocations. Because of this, the model has some prior knowledge to draw upon in Task 4 when it first has the opportunity to select how many resources to assign to the leading category.

As mentioned above, this instance-based model has the same structure as the model of probability adjustment. Representation of a trial instance consists of three parts: a decision context (in this case, the probability of the leading category), the decision itself (i.e., the resource allocation to the leading category), and the outcome of the decision (i.e., the payoff resulting from the match of that allocation to the ground truth of the identity of the responsible category). This representation is natural because all these pieces of information are available during a resource allocation instance and can plausibly be bound together in episodic memory. However, the problem is how to leverage it to make decisions.

Decision-making (choice) models based on this instance-based learning approach iterate through a small number of possible decisions, generating outcome expectancies from the match of context and decision, and then choose the decision with the highest expected outcome [47, 48]. Control models apply the reverse logic: given the current context and a goal (outcome) state, they match context and outcome to generate the expected action (usually a control value from a continuous domain) that will get the state closest to the goal [49, 50]. However, our problem does not fit either paradigm: unlike choice problems, it does not involve a small number of discrete actions but rather a range of possible allocation values, and, unlike control problems, there is no known goal state (expected outcome) to be reached.

Our model's control logic takes a more complex hybrid approach, involving two steps of access to experiences in declarative memory rather than a single one. The first step consists of generating an expected outcome weighted over the available decisions given the current context. The second step will then generate the decision that most likely leads to that outcome given to the context. Note that this process is not guaranteed to generate optimal decisions, and indeed people do not. Rather, it represents a parsimonious way to leverage our memory of past decisions in this paradigm that still provides functional behavior. A significant theoretical achievement of our approach is that it unifies control models and choice models in a single decision-making paradigm.

When determining how many resources to apply to the lead category, the model initially has only the probability assigned to that category. The first step is to estimate an expected outcome. This is done by performing a blended retrieval on chunks representing past resource allocation decisions using the probability as a cue. The outcome value of the retrieved chunk is the expected outcome for the trial. Next, based on the probability assigned to the leading category and the expected outcome, an additional blended retrieval is performed. The partial matching mechanism is leveraged to allow for nonperfect matches to contribute to the estimation of expected outcome and resource quantity. The resource allocation value of this second blended allocate chunk is the quantity of resources that the model assigns to the leading category. After feedback is received, the model learns a resource allocation decision chunk that associates the leading category probability, the quantity of resources assigned to the leading category, and the actual outcome of the trial (i.e., the resource allocation score for that trial). Additionally, up to two counterfactual chunks are committed to declarative memory. The counterfactuals represent what would have happened if a winner-take-all resource assignment had been applied and what would have happened if a pure probability-matched resource assignment (i.e., using the same values as the final probabilities) had been applied. The actual nature of the counterfactual assignments is not important; what is essential is to give the model a broad enough set of experience representing not only the choices made but also those that could have been made.

The advantage of this approach is that the model is not forced to choose between a discrete set of strategies such as winner-take-all or probability matching; rather, various strategies could emerge from instance-based learning. By priming the model with the winner-take-all and probability matching strategies (essentially the boundary conditions), it is possible for the model to learn any strategy in between them, such as a tendency to more heavily weigh the leading candidate (referred to as PM+), or even suboptimal strategies such as choosing 25% for each of the four categories (assuring a score of 25 on the trial) if the model is unlucky enough to receive enough negative feedback so as to encourage risk aversion [47].

#### 3.3.5. Layer Selection

Layer selection in Task 6 depends on learning the utilities of layer choices in Tasks 4-5 and relies on four processes: instance-based learning (similar to probability adjustment and resource allocation mechanisms), difference reduction heuristic, reinforcement learning, and cost-satisfaction. During Tasks 4–6 participants were asked to update probability distributions based on the outcome of each layer (i.e., feature and relevant decision rule). In Tasks 4-5, participants experienced 20 instances of the SOCINT rule and 10 instances each of the IMINT, MOVINT, and SIGINT rules. They also had a variable number of instances from Task 6 based on their layer selections. Some of the layers and outcomes might support their preferred hypothesis, but some of them might not. Based on the results of each layer's outcome, the gain towards the goal of identifying a single category might vary, and those experiences affect future layer selection behavior through reinforcement learning.

A rational Bayesian approach to Tasks 4–6 might involve the computation of expected information gains (EIGs) computed overall possible outcomes that might result from the selection of a feature layer. Under such a rational strategy, SIGINT and SOCINT layers would require more calculation cost than IMINT and MOVINT. In particular, the calculation of EIG for SIGINT requires considering four categories with two outcomes each, and the calculation of EIG for SOCINT requires four outcomes; however, the calculation of EIG for an IMINT or MOVINT layer requires consideration of only two outcomes. We assume participants might consider the cognitive costs of exploring layer selection outcomes in preferring certain layer selection.

We chose to use a difference reduction heuristic (i.e., hill-climbing) because we assume that an average person is not able to compute and maintain the expected information gain for all layers. A hill-climbing heuristic enables participants to focus on achieving states that are closer to an ideal goal state with the same requirement for explicit representation, because all that needs to be represented is the difference between the current state and a preferred (i.e., goal) state.

In Task 6, all prior instances were used to perform evaluations of layer selection. First, the model attends to the current problem state, including the distribution of likelihood of attacks, as represented in the goal (Prefrontal Cortex; PFC) and imaginal (Parietal Cortex; PC) buffers. Then, the model attempts to retrieve a declarative memory chunk (Hippocampus/Medial Temporal Lobe; HC/MTL) which encodes situation-action-outcome-utility experiences of past layer selections. This mechanism relies on partial matching to retrieve the chunks that best match the current goal situation and then on blending to estimate the utility of layer-selection moves based on past utilities. If the retrieval request fails, then the model computes possible layer-selection moves (i.e., it performs a look-ahead search) using a difference-reduction problem-solving heuristic. In difference reduction, for each mentally simulated layer-selection action, the model simulates and evaluates the utility of the outcome (with some likelihood of being inaccurate). Then the model stores a situation-action-outcome-utility chunk for each mentally simulated move. It is assumed that the number of moves mentally searched will not often be exhaustive. This approach is similar to the use of counterfactuals in the resource allocation model.

The ACT-R model of Task 6 relies on the use of declarative chunks that represent past Tasks 4, 5, and 6 experiences. This is intended to capture a learning process whereby participants have attended to a current probability distribution, chosen a layer, revised their estimates of the hypotheses, and finally assessed the utility of the layer selection they just made. The model assumes that chunks are formed from these experiences each representing the specific situation (probability distribution over groups), selected intelligent layer, and observed intelligence outcome and information utility, where the utilities are computed by the weighted distance metric (*d*) below:

(4) $$\begin{array}{c} d=\overset{}{\underset{i\in \text{Hypotheses}}{\sum }}p_{i}\left(1-p_{i}\right), \end{array}$$

where, each *p* is a posterior probability of a group attack based on rational calculation, and zero is the optimum. The ACT-R model uses this weighted distance function and assumes that the participant's goal is to achieve certainty on one of the hypotheses (i.e., *p*_*i*_ = 1).

At a future layer selection point, a production rule will request a blended/partial matching retrieval from declarative memory based on the current situation (probability distribution over possible attacking groups). ACT-R will use a blended retrieval mechanism to partially match against previous experience chunks and then blend across the stored information utilities for each of the available intelligence layer choices. For each layer, this blending over past experience of the information utilities will produce a kind of expected information utility for each type of intelligence for specific situations. Finally, the model compares the expected utility of different intelligence layers and selects the one with the highest utility.

The ACT-R model performs reinforcement learning throughout Tasks 4 to 6. After updating probability distribution based on a layer and its outcomes, the model evaluates whether it has gained or lost information by comparing the entropy of the prior distribution with the posterior distribution. If it has gained information, the production for the current layer receives a reward if it has lost, it receives a punishment. This reinforcement learning enables the model to acquire a preference order for the selection of intelligence layers, and this preference order list was used to determine which layer should be explored first in the beginning of the layer selection process.

### 3.4. Cognitive Biases Addressed

Anchoring and confirmation biases have been long studied in cognitive psychology and the intelligence communities [9, 51–55]. As we have already mentioned, these biases emerge in several ways in the ACT-R model of AHA tasks (see Table 4 for an overview). In general, our approach accounts for three general sources of biases.

The first source of bias is the architecture itself, both in terms of mechanisms and limitations. In our model, a primary mechanistic source of bias is the ACT-R blending mechanism that is used to make decisions by drawing on instances of similar past problems. While it provides a powerful way of aggregating knowledge in an efficient manner, its consensus-driven logic tends to yield a regression to the mean that often (but not always) results in an anchoring bias. Limitations of the architecture such as working memory capacity and attentional bottlenecks can lead to ignoring some information that can result in biases such as base-rate neglect (ignoring background frequencies when making a judgment of conditional probabilities).

The second source of bias is the content residing in the architecture, most prominent strategies in the form of procedural knowledge. Strategies can often lead to biases when they take the form of heuristic that attempt to conserve a limited resource, such as only updating a subset of the probabilities in order to save time and effort, or overcome a built-in architectural bias, such as the centroid-of-centroid strategy intended to combat the recency effect in chunk activations that in turn leads to an anchoring bias.

The third and final source of biases is the environment itself, more specifically its interaction with the decision-maker. For instance, the normalization functionality in the experimental interface can lead to anchoring bias if it results in a double normalization. Also, the feedback provided by the environment, or lack thereof, can lead to the emergence or persistence of biases. For instance, the conservatism bias that is often seen in the generation of probabilities could persist because subjects do not receive direct feedback as to the accuracy of their estimates.

In the next subsections we discuss in detail the sources of the various biases observed.

#### 3.4.1. Anchoring and Adjustment

Anchoring is a cognitive bias that occurs when individuals establish some beliefs based on some initial evidence and then overly rely on this initial decision in their weighting of new evidence [54]. Human beings tend to anchor on some estimates or hypotheses, and subsequent estimates tend to be adjustments that are influenced by the initial anchor point—they tend to behave as if they have an anchoring + adjustment heuristic. Adjustments tend to be insufficient in the sense that they overweight the initial estimates and underweight new evidence.

Anchoring and adjustment in learning (AL) can occur in the first three tasks due to the nature of the task, specifically the iterative generation of the centroids of each category across each trial. For each trial, participants estimate a centroid for the events, perceived to date by that category, then observe a new set of events and issue a revised estimate. This process of issuing an initial judgment and then revising might lead to anchoring and adjustment processes. Thus, in Tasks 1–3, anchoring can occur due to the centroid-of-centroid strategy to prevent being overly sensitive to the most recent events.

Tasks 4–6 can also elicit anchoring biases. Anchoring bias in weighing evidence might be found when participants revise their belief probabilities after selecting and interpreting a particular feature. The estimates of belief probabilities that were set prior to the new feature evidence could act as an anchor, and the revised (posterior) belief probabilities could be insufficiently adjusted to reflect the new feature (i.e., when compared to some normative standards). Insufficient adjustment may happen because the blended retrieval mechanism tends to have a bias towards the mean.

The model also updates only the probabilities corresponding to the positive outcomes of the decision rules. For example, if it is discovered that the probe occurs on a major road, the model would update the probabilities for categories A and B and neglect to adjust downward the probabilities for categories C and D. This neglect is assumed to result from a desire to save labor by relying on the interface normalization function and by the difficulty of carrying out the normalization computations mentally. In turn, this is a cause of an underestimation of probabilities (anchoring) that results from the normalization of the probabilities in the interface.

#### 3.4.2. Confirmation Bias

Confirmation bias is typically defined as the interpretation of evidence in ways that are partial to existing beliefs, expectations, or a hypothesis in hand [53], the tendency for people to seek information and cues that confirm the tentatively held hypothesis or belief and not seek (or discount) those that support an opposite conclusion or belief [56], or the seeking of information considered supportive of favored beliefs [53]. Studies [57–59] have found evidence of confirmation bias in tasks involving intelligence analysis, and there is a common assumption that many intelligence failures are the result of confirmation bias in particular [9, 60].

Confirmation bias in weighing evidence might occur in the probability adjustment process in Tasks 4–6. For example, we have seen that the probability adjustment process applied to small probability values sometimes resulted in over-adjustment. Certain strategies for probability adjustment might also result in confirmation bias. When applying a particular feature, such as IMINT (which supports two hypotheses), participants may only apply the adjustment to the preferred hypothesis while neglecting the other category that is also supported by evidence or weight the evidence too strongly in favor of the preferred category.

Confirmation bias can also occur in the evidence seeking process in Task 6 as the participants might select intelligence layers that maximize information gains about the current preferred category. For instance, when applying the IMINT and MOVINT rules, one could only apply the adjustment to the preferred hypothesis (assuming it is one of the two receiving favorable evidence from that layer) while neglecting the other categories also supported by the evidence. This strategic decision could reflect the desire both to minimize effort and to maximize information gain.

A significant difference in layer selection features is the SIGINT feature, which requires the selection of one particular category to investigate. If that feature is applied to the leading category and chatter is detected, then the category likelihood gains considerable weight (by a factor of 7). However, if no chatter is detected, then the category likelihood is strongly downgraded, which throws considerable uncertainty over the decision process. Thus the decision to select the SIGINT layer too early (before a strong candidate has been isolated) or to apply it to strictly confirm the leading category rather than either confirm or invalidate a close second might be construed as a form of confirmation bias in layer selection.

#### 3.4.3. Base-Rate Neglect

Base-rate neglect is an error that occurs when the conditional probability of a hypothesis is assessed without taking into account the prior background frequency of the hypothesis' evidence. Base-rate neglect can come about from three sources.

1. Higher task difficulties and more complex environments can lead to base-rate neglect due to the sheer volume of stimuli to remember. To reduce memory load, some features may be abstracted.
2. Related to the above, there can be base-rate neglect due to architectural constraints. For instance, short-term memory is generally seen to have a capacity of 7 ± 2 chunks of information available. Once more chunks of information need to be recalled, some information may either be abstracted or discarded.
3. Finally, there can be explicit knowledge-level strategic choices made from an analysis of (1) and (2) above.

The strategic choice (3) of the ACT-R model leads to base-rate neglect in calculating probabilities for Tasks 1–3. In particular, the fact that the ACT-R model generates probabilities based on category centroids leads to base-rate neglect. This is because base-rate information is not directly encoded within the category centroid chunk. The information is still available within the individual SIGACTs stored in the model, but it is not used directly in generating probabilities.

#### 3.4.4. Probability Matching

We endeavored to develop a model that leveraged subsymbolic mechanisms that often give rise naturally to probability matching phenomena [61]. Subsymbolic mechanisms in ACT-R combine statistical measures of quality (chunk activation for memory retrieval, production utility for procedural selection) with a stochastic selection process, resulting in behavior that tends to select a given option proportionately to its quality rather than in a winner-take-all fashion. This approach is similar to stochastic neural models such as the Boltzmann Machine [35].

In our model, resource allocation decisions are based not on discrete strategies but rather on the accumulation of individual decision instances. Strategies then are an emergent property of access to those knowledge bases. Moreover, to unify our explanation across biases, we looked to leverage the same model that was used to account for anchoring (and sometimes confirmation) bias in probability adjustment.

Results also approximate those seen by human participants: a wide variation between full probability matching and winner-take-all, several individual runs tending towards uniform or random distributions, and the mean falling somewhere between probability matching and winner-take-all (closer to matching).

Probability matching in resource allocation occurs due to the trade-off inherent in maximizing reward versus minimizing risk. A winner-take-all is the optimal strategy overall; however there are individual trials with large penalties (a zero score) when a category other than the one with the highest probability is the ground truth. When such an outcome occurs prominently (e.g., in the first trial), it can have a determinant effect on subsequent choices [47].

## 4. Data and Results

The results of the ACT-R model on the AHA tasks were compared against 45 participants who were employees of the MITRE Corporation. All participants completed informed consent and debriefing questionnaires that satisfied IRB requirements. To compare the ACT-R model to both human participants and a fully Bayesian rational model, several metrics were devised by MITRE [62–64], which are summarized below.

As an overall measure of uncertainty across a set of hypotheses, we employed a *Negentropy* (normalized negative entropy) metric, *N*, computed as

(5) $$\begin{array}{c} N=\frac{\left(E_{\max}-E\right)}{E_{\max}}, \end{array}$$

where *E* is the Shannon entropy computed as

(6) $$\begin{array}{c} E=-\overset{}{\underset{h}{\sum }}P_{h}*\log_{2}P_{h}, \end{array}$$

where the summation is over the probabilities, *P*_*h*_, assigned to hypotheses. Negentropy can be expressed on a scale of 0% to 100%, where *N* = 0% implies maximum uncertainty (i.e., maximum entropy or a uniform probability distribution over hypotheses) and *N* = 100% implies complete certainty (i.e., zero entropy or a distribution in which one hypothesis is assigned a maximum probability of 1). The normalization is provided by *E*_max⁡_ = 2 in the case of four hypotheses (Tasks 2–6) and *E*_max⁡_ = 1 in the case of two hypotheses (Task 1).

Comparisons of human and normative (e.g., Bayesian) assessments of certainty as measured by Negentropy permit the evaluation of some cognitive biases. For instance, one can compare human Negentropy *N*_*H*_ to Negentropy for a rational norm *N*_*Q*_ following the revision of probabilities assigned to hypotheses after seeing new intelligence evidence. If *N*_*H*_>*N*_*Q*_, then the human is exhibiting a confirmation bias because of overweighing evidence that confirms the most likely hypothesis. On the other hand, if *N*_*H*_ < *N*_*Q*_, then the human is exhibiting conservatism which might arise from an anchoring bias.

In addition to measuring biases, we also compared the probability estimation and resource allocation functions of the model against both human participants and a Bayesian rational model (i.e., an optimal model). The Kullback-Leibler Divergence (*K*) is a standard information-theoretic measure for comparing two probability distributions like those of a human (*P*) and model (*M*). *K*_*PM*_ measures the amount of information (in bits) by which the two distributions differ, which is computed as follows:

(7) KPM=EPM−EP  =−∑hPh∗log⁡2⁡Mh+  ∑hPh∗log⁡2⁡Ph,

where, similar to the Negentropy measure, *E*_*PM*_ is the cross-entropy of human participants (*P*) and the ACT-R model (*M*) and *E*_*P*_ is the entropy of human participants. It is important to note that *K*_*PM*_ = 0 when both distributions are the same, and *K*_*PM*_ increases as the two distributions diverge. *K* ranges from zero to infinity, but *K* is typically less than 1 unless the two distributions have large peaks in different hypotheses.

A normalized measure of similarity (*S*) on a 0–100% scale similar to that of Negentropy can be computed from *K*:

(8) $$\begin{array}{c} S=100\%*2^{-K}. \end{array}$$

As the divergence *K* ranges from zero to infinity, the similarity *S* ranges from 100% to 0%. Thus *S*_*QP*_ and *S*_*QM*_ can be useful for comparing the success of humans or models in completing the task (compared by their success relative against a fully rational Bayesian model). This measure will be referred to as an S1 score.

To address the overall fitness of the model output compared with human data, the most direct measure would be a similarity comparing the human and model distributions (*S*_*PM*_) directly. However, this would not be a good measure as it would be typically higher than 50% (*K* is typically less than 1); thus we scaled our scores on a relative basis by comparing against a null model. A null model (e.g., a uniform distribution, *R* = {0.25, 0.25, 0.25, 0.25}) exhibits maximum entropy, which implies “random” performance in sensemaking. Thus *S*_*PR*_ was used to scale as a lower bound in computing a relative success rate (RSR) measure as follows:

(9) $$\begin{array}{c} \text{RSR}\;=\frac{\left(S_{PM}-S_{PR}\right)}{\left(100\%-S_{PR}\right)}. \end{array}$$

The model's RSR was zero if *S*_*PM*_ is equal to or less than *S*_*PR*_, because in that case a null model *R* would provide the same or better prediction of the human data as the model. The RSR for a model *M* will increase as *S*_*PM*_ increases, up to a maximum RSR of 100% when *S*_*PM*_ = 100%. For example, if a candidate model *M* matches the data *P* with a similarity score of *S*_*PM*_ = 80% and the null model *R* matches *P* with a similarity score of *S*_*PR*_ = 60%, then the RSR for model *M* would be (80 − 60)/(100 − 60) = (20/40) = 50%.

In Task 6, because each participant potentially receives different stimuli at each stage of the trial as they choose different INTs to receive, RSR was not appropriate. Instead, the model was assessed against a relative match rate (RMR), which is defined below.

After receiving the common HUMINT feature layer at the start of each trial in Task 6, human participants have a choice amongst four features (IMINT, MOVINT, SIGINT, or SOCINT). The next choice is among three remaining features, and the last choice is among two remaining features. Thus there are 4∗3∗2 = 24 possible sequences of choices that might be made by a subject on a given trial of Task 6. For each trial, the percentage of subjects that chooses each of the 24 sequences was computed. The modal sequence (maximum percentage) was used to define a benchmark (*t*, *s*_max⁡_) for each trial (*t*), where *F* is the percentage of a sequence and *s*_max⁡_ refers to the sequence with maximum *F* for trial *t*. For each trial, the model predicted a sequence of choices *s*_ mod _, and the percentage value of *F*(*t*, *s*_ mod _) for this sequence was computed from the human data. In other words, *F*(*t*, *s*_ mod _) is the percentage of humans that chose the same sequence as the model chose, on a given trial *t*:

(10) $$\begin{array}{c} \text{RMR}\left(t\right)\;=\;\frac{F\left(t,s_{\text{ mod }}\right)}{F\left(t,s_{\max}\right)}. \end{array}$$

For example, assume a model predicts a sequence of choices *s*_ mod_ on a trial of Task 6. Assume also that 20% of human subjects chose the same sequence, but a different sequence was the most commonly chosen by human subjects, for example, by 40% of subjects. In that case *F*(*t*, *s*_ mod _) = 20% and *F*(*t*, *s*_max⁡_) = 40%, so RMR(*t*) = 20%/40% = 50%.

Finally, a measure of resource allocation was derived by assigning a value (S2) based on the resources allocated to the category that was the ground truth. Thus if a participant (or model) was assigned a resource allocation of {A% = 40, B% = 30, C% = 20, D% = 10} and the ground truth was category B, then the S2 score for that trial would be 30%. Thus, to maximize the score, an optimal model or participant would need to assign 100% of their resources to the ground truth (i.e., adopt a winner-take-all strategy to resource allocation).

### 4.1. Data

The integrated ACT-R AHA model performs (and learns incrementally across) all 6 tasks using the same knowledge constructs (production rules and chunks; other than those it learns as part of executing the task) and parameters. The results comparing human and model performance presented below are broken down by task and expanded on trial-by-trial and layer-by-layer analyses. For instance, while a similar instance-based representation is used across all tasks for probability adjustment, resource allocation, and layer selection, the output from the path planning mechanism is only used in Tasks 3 and 4. Thus it is best to examine Task 3 and Task 4-1 (the first layer of Task 4) alone in determining the efficacy of the path planning mechanism.

The model was run the same number of times as participants in the dataset (45) with the average model response were compared to the average human performance. The natural variability in the model (stochastic elements influencing instance-based learning) approximates some of the individual differences of the human participants. While the average distribution of the ACT-R model is slightly peakier than the average human (the ACT-R model is closer to Bayesian rational than humans are), the overall fits (based on RSR/RMR) are quite high, with an overall score over .7 (a score of 1 indicates a perfect fit [63, 64]; see Table 5). In addition, a linear regression comparing model and human performance at each block of each layer indicates that the model strongly and significantly predicts human behavior on AHA tasks.

**Figure 11:**
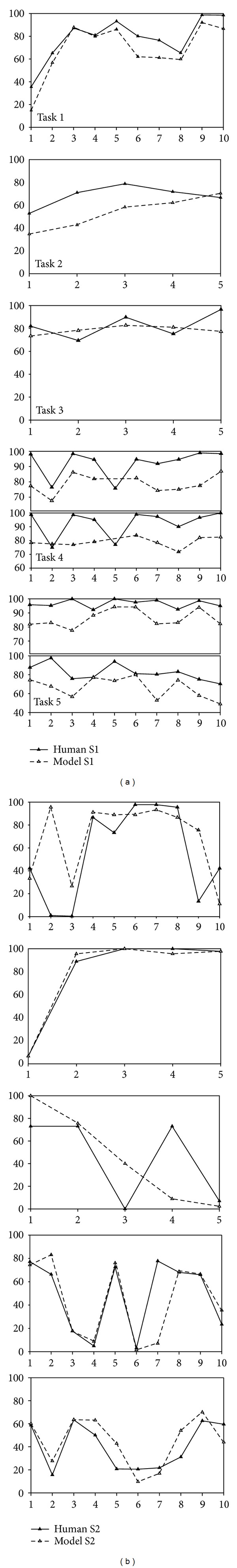
(a) is the trial-by-trial (horizontal axis) fit between the ACT-R model and human data for Tasks 1–5 using the S1 metric (vertical axis), which compares humans and model to Bayesian rational. (b) is the fit for the S2 metric determining resource allocation score. For Tasks 4-5, the top tile is the fit for the first feature layer, and the bottom tile is the fit for the final feature layer.

Supporting these results, the trial-by-trial performance of the model (see Figure 11) predicted many of the variations seen in users' data. While the ACT-R model tended to behave more rationally than human participants (i.e., the model exhibited a higher S1 score), the model tended to capture much of the individual variation of human participants across trials (the S1 scores on Task 2 and S2 scores on Task 3 being the exceptions).

**Figure 12:**
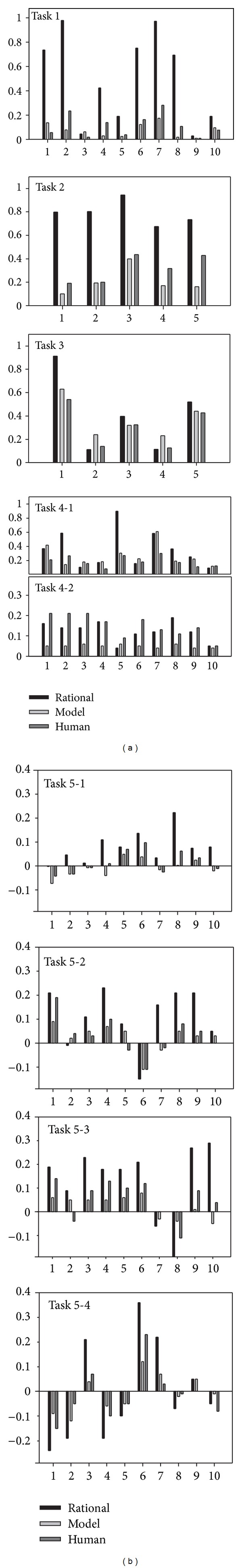
Trial-by-trial Negentropy scores for Tasks 1–5 (Δ Negentropy between layers for Tasks 4-2 and 5) for the fully rational Bayes outcome, the ACT-R model, and human participants. Values less than normative (i.e., Bayesian rational) are considered an anchoring bias, and values greater than normative are considered confirmation bias.

In addition to the fit to human data based on probabilities (S1/RSR) and resource allocation (S2), the model was also compared to human participants in terms of the anchoring and adjustment and confirmation biases (see Figure 12). Whenever both the human behavioral data and model exhibit a lower Negentropy than the Bayesian rational model, they are both exhibiting anchoring bias (and conversely they exhibit confirmation bias when they have a higher Negentropy). As shown below, the ACT-R model significantly predicts not only the presence or absence of a bias but also the quantity of the bias metric, reflected in an overall *R* ^2^ = .645 for Negentropy scores across all tasks.

#### 4.1.1. Tasks 1 and 2

In Task 1, the ACT-R model produces a probability distribution and forced-choice resource allocation for each trial. The probability distribution is based on the blended probability adjustments using instance-based learning as described above and results in an increased prevalence of anchoring (i.e., less peaky distributions) over the normative solution in a manner similar to (yet stronger than) human data.

Similar to Task 1, in Task 2 the model follows the general trends of human participants for both S1 and especially S2 scores. With 4 groups to maintain in Task 2, we assume that there is more base-rate neglect in humans (which results in ACT-R from the centroid representation of groups that loses base-rate information), which increases the RSR score to .799. However, the *R* ^2^ for S1 drops from .929 in Task 1 to .313 in Task 2 because the ACT-R model does not capture the same trial-by-trial variability despite being closer to mean human performance.

In Task 1, the ACT-R model exhibited a mean Negentropy score (*N*_*M*_ = .076), well below that of the Bayesian solution (*N*_*Q*_ = .511); thus, there was an overall strong trend towards anchoring and adjustment in learning (AL) for the model. Humans exhibited a similar AL bias (*N*_*H*_ = .113). Additionally, on a trial-by-trial comparison of the model to the Bayesian solution, both humans and the ACT-R model showed AL for each individual trial.

In Task 2 the results were similar (*N*_*Q*_ = .791, *N*_*M*_ = .206, *N*_*H*_ = .113) with both the model and humans exhibiting anchoring and adjustment in learning in every trial.

#### 4.1.2. Task 3

In Task 3 the ACT-R model was first required to generate category centroids based on a series of events and then was required to use the path planning mechanism to estimate the distance between each category centroid and a probe location. While the model captured average human performance on the task, it was not able to capture individual human behavior. This was in part due to wide variability and negative skew in the raw human data and a difficulty in the ACT-R model correctly placing category centroids when events fell across multiple roads.

However, when examining bias metrics, the ACT-R model exhibited both AL and confirmation biases as did human participants. Both ACT-R and human participants exhibited an AL bias on Trials 1, 3, and 5 and confirmation bias on Trials 2 and 4. Overall, both the model and humans exhibit a similar AL (*N*_*Q*_ = .412, *N*_*M*_ = .372, and  *N*_*H*_ = .311). Thus, while the model was not capturing the exact distance estimates of human participants, it was able to capture the variability in the bias metrics.

#### 4.1.3. Task 4

In Task 4, the issue with centroid generation over multiple roads is avoided since centroids are provided by the task environment, resulting in a HUMINT layer RSR = .761 and *R* ^2^ = .730. Thus, the path-planning mechanism itself is functioning correctly and providing excellent fits to human data. In addition, the model provided excellent fits to the second layer (the SOCINT layer) in Task 4, with an RSR fit of .905.

Beginning with Task 4, layer 2, the measure of anchoring and adjustment (Delta Negentropy) is based on whether category probabilities were revised sufficiently by following the probabilistic decision rules. There was an overall trend towards anchoring and adjustment in both learning and inference, with a slight trend towards confirmation bias for the humans. The main difference is when using SOCINT; the ACT-R model tends to exhibit an anchoring bias while human participants tended to exhibit a confirmation bias when applying the SOCINT layer. We speculate that the reason why humans would exhibit a confirmation bias on SOCINT, which is the easiest of the rules to apply, might be that it has a compelling visual interpretation that participants are more likely to trust.

Also, beginning with Task 4, resource allocation judgments are a distribution instead of a forced-choice. The model learns the outcomes of probability matching (PM) versus winner-take-all (WTA; forced-choice) through experience on Tasks 1–3 in the form of IBL chunks. From this experience, the model adopts a strategy (not a procedural rule but emergent from blended retrieval of chunks) that is somewhere between PM and WTA, with a bias towards PM. Based on the S2 fits for Tasks 4–6 (see Table 5), the resource allocation mechanism, which also relies on the same instance-based learning approach as the probability adjustment mechanism, provides an excellent match to human data.

#### 4.1.4. Task 5

In Task 5 the RSR fits for Layers 1–3 are quite high (.856, .776, and .780, resp.) with some drop-off in Layer 4 (.618) due to human participants' distributions being closer to uniform and an RSR singularity (a near-uniform Bayesian, human, and model distribution leading to all nonperfect fits receiving a near-zero score since the random model near-perfect predicts human behavior). It may also be the case that humans, after getting several pieces of confirmatory and disconfirmatory evidence, express their uncertainty by flattening out their distribution in the final layer rather than applying each layer mechanically.

As seen in the Delta Negentropy graphs for each layer (see Figure 12), ACT-R correctly predicts the overall trend of anchoring (*N*_*H*_ < *N*_*Q*_ and *N*_*M*_ < *N*_*Q*_) for each layer:

 Layer 1: *N*_*q*_ = .080, *N*_*h*_ = .016, *N*_*m*_ = −.007
 Layer 2: *N*_*q*_ = .110, *N*_*h*_ = .033, *N*_*m*_ = .025
 Layer 3: *N*_*q*_ = .138, *N*_*h*_ = .056, *N*_*m*_ = .024
 Layer 4: *N*_*q*_ = .000, *N*_*h*_ = −.007, *N*_*m*_ = −.011

Across each layer, the model correctly predicts anchoring on all 10 trials of Layer 2, correctly predicts anchoring on 8 trials of Layer 3 and correctly predicts the confirmation on the other 2 trials, correctly predicts anchoring on 8 trials of Layer 4 and correctly predicts confirmation on the other 2, and correctly predicts anchoring on 4 trials of Layer 5 and correctly predicts confirmation on 5 other trials. Over 40 possible trials, ACT-R predicts human confirmation and anchoring biases on 39 of the trials (trial 10 of Layer 5 being the only exception).

#### 4.1.5. Task 6

**Figure 13:**
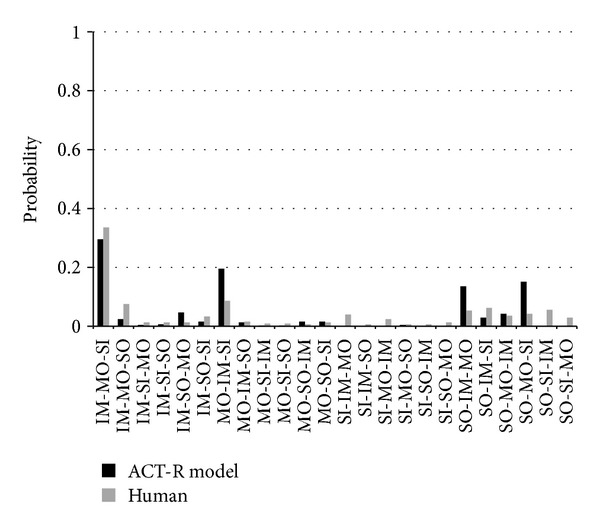
Layer selection sequences both the ACT-R model and human data (IM for IMINT, MO for MOVINT, SI for SIGINT, and SO for SOCINT).

In Task 6, both the model and participants are able to choose 3 feature layers before specifying a final probability distribution.  Figure 13 shows the probability distribution of layer selection sequences for our ACT-R model and human data. To measure the similarity of the probability distribution of layer selection sequences between the ACT-R model and human data, we performed Jensen-Shannon divergence analysis, which is a method of measuring the similarity between two distributions. The divergence between the two distributions is .35, indicating that the ACT-R model strongly predicts the human data patterns.

## 5. Generalization

**Figure 14:**
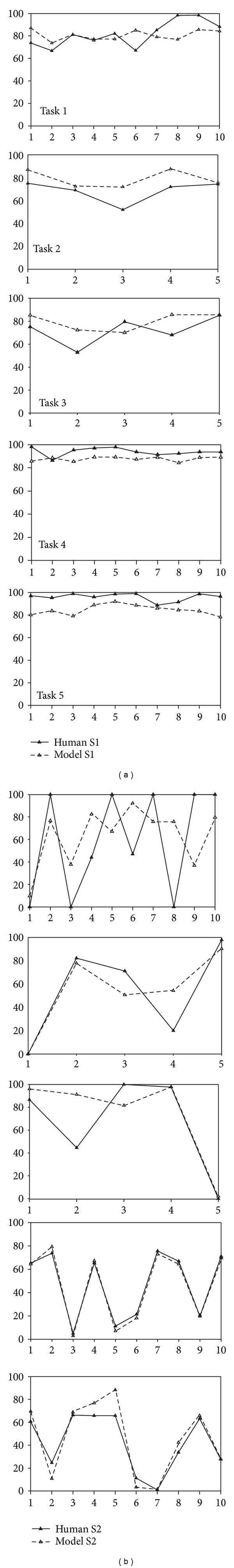
(a) is the trial-by-trial fit between the ACT-R model and human data for Tasks 1–5 using the S1 metric, which compares humans and model to Bayesian rational. (b) is the fit for the S2 metric determining resource allocation score. For Tasks 4-5, the graph represents the final layer fit. These results are for the final Penn State dataset.

To determine the generalizability of the integrated ACT-R model of the AHA tasks, the same model that produced the above results was run on novel stimuli in the same AHA framework. The results of the model were then compared to the results of a novel sample gathered from 103 students at Penn State University. This new data set was not available before the model was run, and no parameters or knowledge structures were changed to fit this data set. Unlike the original 45-participant dataset, the Penn State sample used only people who had taken course credit towards a graduate Geospatial Intelligence Certificate. Overall, the RSR and *R* ^2^ fits on S2 scores improved while the *R* ^2^ fits on S1 scores dropped (see Table 6). The increase in RSR was mainly due to the Penn State population behaving more rationally (i.e., higher S1 scores; see Figure 14) than the population from the initial dataset. This is consistent with the increased education and experience of the Penn State sample. That said, the Penn State sample most likely utilized some different strategies in solving the tasks, as the trial-by-trial S1 fits were not as close, implying some difference in reasoning that the ACT-R model was not capturing.

Overall, the improved model fits indicate that the ACT-R model of the AHA tasks is able to capture average human performance at the task level for S1 scores and at the trial-by-trial level for S2 scores. Conversely, this justifies the reliability of the AHA tasks as a measure of human performance in a sensemaking task environment.

**Figure 15:**
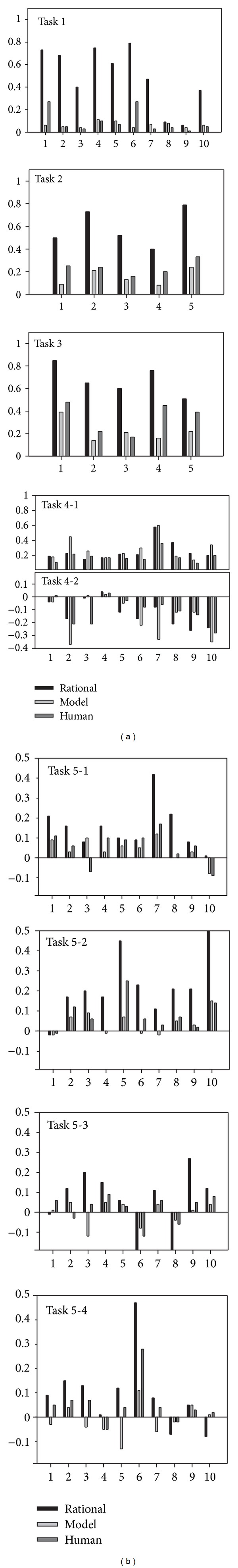
Trial-by-trial Negentropy scores for Tasks 1–5 (Δ Negentropy between layers for Tasks 4-2 and 5) for the fully rational Bayes outcome, the ACT-R model, and human participants. These results are for the Penn State dataset.

Finally, the ACT-R model fits for anchoring and confirmation biases (see Figure 15) were also similar in the Penn State dataset. The model correctly predicted both the presence and degree of anchoring on every block in Tasks 1–3 and followed similar trial-by-trial trends for both anchoring and confirmation in Tasks 4-5. *R* ^2^ of Negentropy scores was a similar .591 to the original dataset.

## 6. Conclusion

The decision-making literature has established a lengthy list of cognitive biases under which human decision making empirically deviates from the theoretical optimum. Those biases have been studied in a number of theoretical (e.g., binary choice paradigms) and applied (e.g., medical diagnosis and intelligence analysis) settings. However, as the list of biases and experimental results grows, our understanding of the mechanisms producing these biases has not followed pace. Biases have been formulated in an ad hoc, task- and domain-specific manner. Explanations have been proposed ranging from the use of heuristic to innate individual preferences. What is lacking is an explicit, unified, mechanistic, and theoretical framework for cognitive biases that provides a computational understanding of the conditions under which they arise and of the methods by which they can be overcome.

In this paper, we present such a framework by developing unified models of cognitive biases in a computational cognitive architecture. Our approach unifies results along a pair of orthogonal dimensions. First, the cognitive architecture provides a functional computational bridge from qualitative theories of sensemaking to detailed neural models of brain functions. Second, the framework enables the integration of results across paradigms from basic decision making to applied fields. Our basic hypothesis is that biases arise from the interaction of three components: the task environment, including the information and feedback available as well as constraints on task execution, the cognitive architecture, including cognitive mechanisms and their limitations, and the use of strategies including heuristic as well as formal remediation techniques. This approach unifies explanations grounded in neurocognitive mechanisms with those assuming a primary role for heuristic. The goal is to derive a unified understanding of the conditions under which cognitive biases appear as well as those under which they can be overcome or unlearned. To achieve this unification, our model uses a small set of architectural mechanisms, leverages them using a coherent task modeling approach (i.e., instance-based learning), performs a series of tasks using the same knowledge structures and parameters, generalizes across different sets of scenarios and human participants, and quantitatively predicts a number of cognitive biases on a trial-to-trial basis.

In particular, we show biases to be prevalent under system 1 (automatic) processes [65] and that a unified theory of cognition can provide a principled way to understand how these biases arise from basic cognitive and neural substrates. As system 2 (deliberative) processes make use of the same cognitive architecture mechanisms in implementing access to knowledge and use of strategies, we expect biases to also occur, in particular as they relate to the interaction between the information flow and the structure of the architecture. However, at the same time, we show that system 2 processes can provide explicit means to remediate most effects of the biases, such as in the centroid-of-centroid generation strategy, where a strong recency bias is replaced with an (slight) anchoring bias.

Moreover, it is to be expected that a rational agent learns and adapts its strategies and knowledge, its metacognitive control (e.g., more deliberate processing of information), and its use of the task environment (e.g., using tools to perform computations or serve as memory aids) so as to at least reduce the deteriorating effects of these biases. However, biases are always subjective, in that they refer to an implicit assumption about the true nature of the world. For instance, the emergence of probability matching in the later tasks can be seen as a response to the uncertainty of the earlier tasks and the seemingly arbitrary nature of the outcomes. Thus, people respond by hedging their bets, a strategy that can be seen as biased in a world of exact calculations but one that has shown its robust adaptivity in a range of real-world domains such as investing for retirement. As is often the case, bias is in the eye of the beholder.

## Figures and Tables

**Figure 16:**
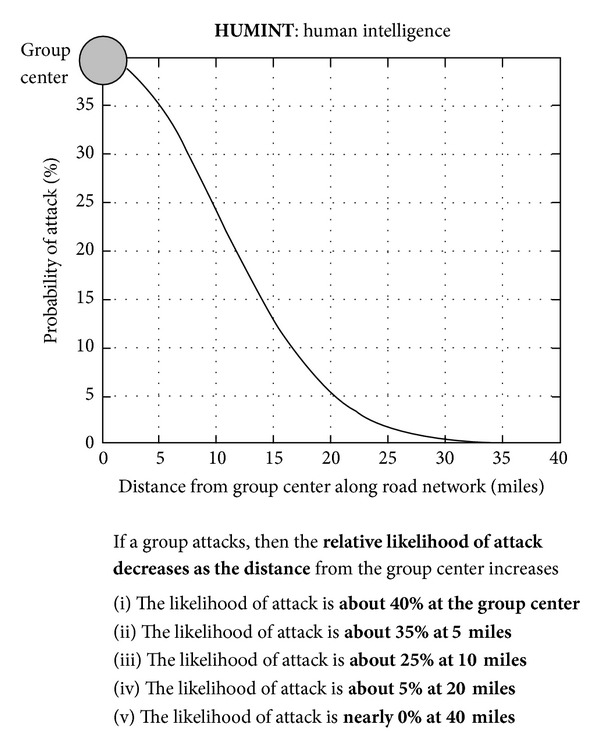
The HUMINT feature, representing distance along a road network between a category and a probe event.

**Figure 17:**
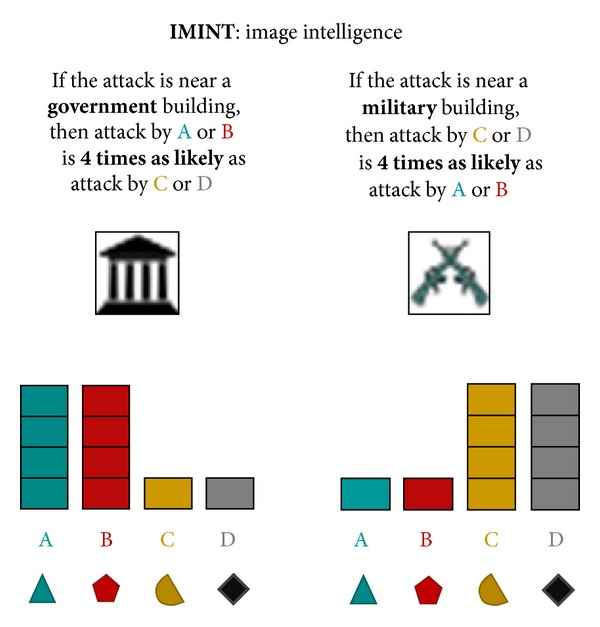
The IMINT feature, representing imagery of government or military buildings located at a probe event location.

**Figure 18:**
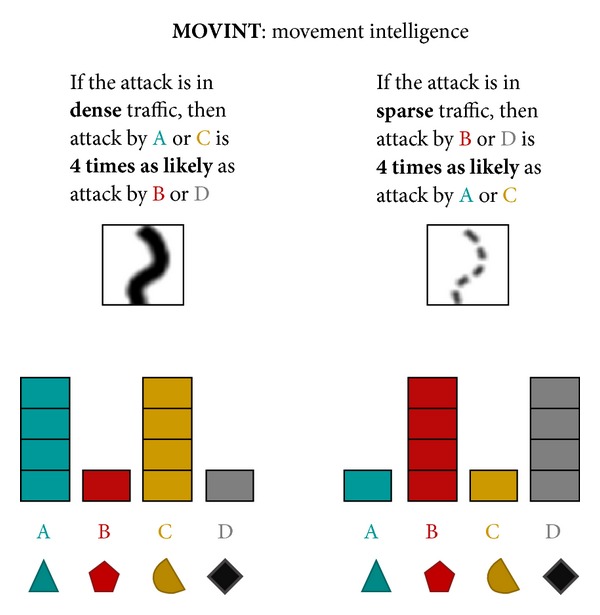
The MOVINT feature, representing vehicular movement information located at a probe event location.

**Figure 19:**
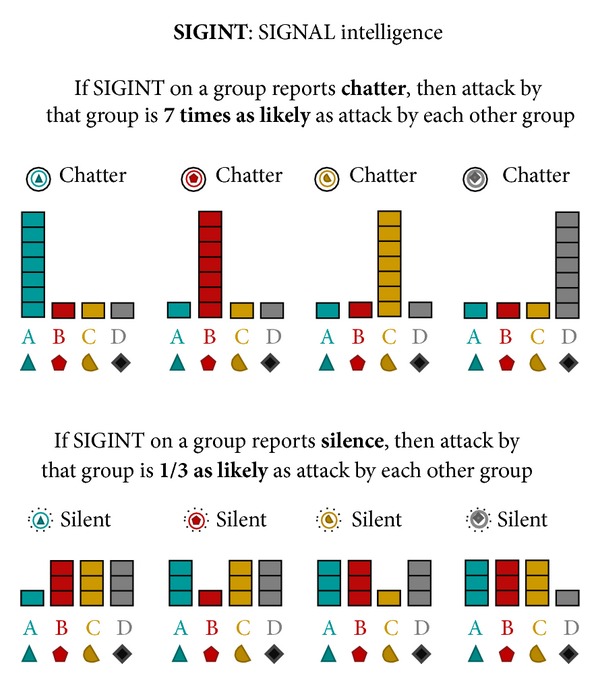
The SIGINT feature, representing chatter located at a probe event location. Note that SIGINT must be picked for a specific category.

**Figure 20:**
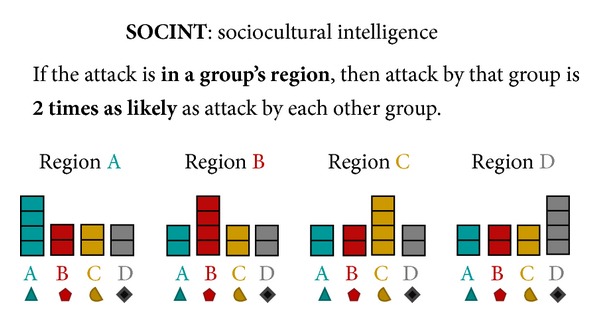
The SOCINT feature, representing sociocultural information about the region boundary for each category.

**Table 1:** Rules for inferring category likelihoods based on knowledge of category centroid location and an observed feature.

Features	Rules
HUMINT	If an unknown event occurs, then the likelihood of the event belonging to a given category decreases as the distance from the category centroid increases.

IMINT	If an unknown event occurs, then the event is four times more likely to occur on a *Government* versus *Military* building if it is from category A or B. If an unknown event occurs, then the event is four times more likely to occur on a *Military* versus *Government* building if it is from category C or D.

MOVINT	If an unknown event occurs, the event is four times more likely to occur in *dense* versus *sparse* traffic if it is from category A or C. If an unknown event occurs, the event is four times more likely to occur in *sparse* versus *dense* traffic if it is from category B or D.

SIGINT	If SIGINT on a category reports *chatter*, then the likelihood of an event by that category is seven times as likely as an event by each other category. If SIGINT on a category reports *silence*, then the likelihood of an event by that category is one-third as likely as an event by each other category.

SOCINT	If an unknown event occurs, then the likelihood of the event belonging to a given category is twice as likely if it is within that category's boundary (represented as a colored region on the display).

**Table 2:** The list of sub-symbolic mechanisms in the ACT-R architecture.

Mechanism	Equation	Description
Activation	*A*_*i*_ = *B*_*i*_ + *S*_*i*_ + *P*_*i*_ + *ε*_*i*_	*B*_*i*_: base-level activation reflects the recency and frequency of use of chunk *i* *S*_*i*_: spreading activation reflects the effect that buffer contents have on the retrieval process *P*_*i*_: partial matching reflects the degree to which the chunk matches the request *ε*_*i*_: noise value includes both a transient and (optional) permanent components (permanent component not used by the integrated model)

Base level	$B_{i}=\ln\left(\overset{n}{\underset{j=1}{\sum }}t_{j}^{-d}\right)+\beta_{i}$	*n*: the number of presentations for chunk *i* *t*_*j*_: the time since the *j*th presentation *d*: a decay rate (not used by the integrated model) *β*_*i*_: a constant offset (not used by the integrated model)

Spreading activation	$S_{i}=\overset{}{\underset{k}{\sum }}\overset{}{\underset{j}{\sum }}W_{kj}S_{ji},$	*k*: weight of buffers summed over are all of the buffers in the model *j*: weight of chunks which are in the slots of the chunk in buffer *k* *W*_*kj*_: amount of activation from sources *j* in buffer *k* *S*_*ji*_: strength of association from sources *j* to chunk *i *
	*S*_*ji*_ = *S* − ln⁡⁡(fan_*ji*_)	*S*: the maximum associative strength (set at 4 in the model) fan_*ji*_: a measure of how many chunks are associated with chunk* j *

Partial matching	$P_{i}=\overset{}{\underset{k}{\sum }}PM_{ki}$	*P*: match scale parameter (set at 2) which reflects the weight given to the similarity *M*_*ki*_: similarity between the value *k* in the retrieval specification and the value in the corresponding slot of chunk *i* The default range is from 0 to −1 with 0 being the most similar and −1 being the largest difference

Declarative retrievals	$P_{i}=\frac{e^{A_{i}/s}}{\sum_{j}^{}e^{A_{j}/s}}$	*P*_*i*_: the probability that chunk *i* will be recalled *A*_*i*_: activation strength of chunk *i* ∑*A*_*j*_: activation strength of all of eligible chunks *j* *s*: chunk activation noise

Blended retrievals	$V=\underset{}{\min}\overset{}{\underset{i}{\sum }}P_{i}{\left(1-{\text{Sim}}_{ij}\right)}^{2}$	*P*_*i*_: probability from declarative retrieval Sim_*ij*_: similarity between compromise value *j* and actual value *i*

Utility learning	*U*_*i*_(*n*) = *U*_*i*_(*n* − 1) + α[*R*_*i*_(*n*) − *U*_*i*_(*n* − 1)],	*U*_*i*_(*n* − 1): utility of production *i *after its *n* − 1st application *R*_*i*_(*n*): reward production received for its *n*th application *U*_*i*_(*n*): utility of production *i* after its *n*th application
	$P_{i}=\frac{e^{U_{i}/s}}{\sum_{j}^{}e^{U_{j}/s}}$	*P*_*i*_: probability that production *i* will be selected *U*_*i*_: expected utility of the production determined by the utility equation above *U*_*j*_: is the expected utility of the competing productions *j *

**Table 3:** Overview of Cognitive Functions of ACT-R Model.

Cognitive function	Overview of operation
Centroid generation Tasks: 1–3	Buffers implicated: blending, imaginal, and goal Biases instantiated: base-rate neglect, anchoring and adjustment The model generates a category centroid by aggregating overall of the perceived events (SIGACTs) in memory via the blended memory retrieval mechanism. Judgments are based on generating a centroid-of-centroids by performing a blended retrieval over all previously generated centroids, resulting to a tendency to anchor to early judgments. Because there is an equal number of centroids per category, this mechanism explicitly neglects base rate

Path planning Tasks: 3-4	Buffers implicated: retrieval, imaginal, and goal Biases instantiated: anchoring and adjustment The model parses the roads into a set of intersections and road segments. The model hill-climbs by starting at the category centroid and appends contiguous road segments until the probe event is reached. Road segment lengths are perceived veridically; however, when recalled the lengths are influenced by bottom-up perceptual mechanisms (e.g., curve complexity and length) simulated by a power law with an exponent less than unity. This leads to underestimation of longer and curvier segments, resulting in a tendency to anchor when perceiving long segments

Probability adjustment Tasks: 1–6	Buffers implicated: blending, imaginal, and goal Biases instantiated: anchoring in weighing evidence, confirmation bias The model represents the prior probability and multiplicative factor rule and then attempts to estimate the correct posterior by performing a blended retrieval over similar chunks in memory in a form of instance-based learning. The natural tendency towards regression to the mean in blended retrievals leads to anchoring bias in higher probabilities and confirmation bias in lower probabilities. The partial matching mechanism is used to allow for matches between the prior and similar values in DM

Resource allocation Tasks: 1–6	Buffers implicated: blending, imaginal, and goal Biases instantiated: probability matching The model takes the probability assigned to a category and then estimates an expected outcome by performing a blended retrieval using the probability as a cue. The outcome value of the retrieved chunk is the expected outcome for the trial. Next, an additional blended retrieval is performed based on both the probability and expected outcome, whose output is the resources allocation After feedback, the model stores the leading category probability, the resources allocated, and the actual outcome of the trial. Up to two counterfactuals are learned, representing what would have happened if a winner-take-all or pure probability matching resources allocation had occurred. Negative feedback on forced winner-take-all assignments in Tasks 1–3 leads to probability matching in Tasks 4–6

Layer selection Task: 4–6	Buffers implicated: blending, goal Biases instantiated: confirmation bias In Task 6, the model uses partial matching to find chunks representing past layer-selection experiences that are similar to the current situation (the distribution of probabilities over hypotheses). If that retrieval succeeds, the model attempts to estimate the utility of each potential layer choice by performing a blended retrieval over the utilities of past layer-choice outcomes in similar situations. The layer choice that has the highest utility is selected. If the model fails to retrieve past experiences similar to the current situations, it performs a “look-ahead” search by calculating the expected utility for some feature layers. The number of moves mentally searched will not often be exhaustive The blended retrieval mechanism will tend to average the utility of different feature layers based on prior experiences from Tasks 4 and 5 (where feature layers were provided to participants), in addition to prior trials on Task 6.

**Table 4:** Source of cognitive biases in the ACT-R integrated model of the AHA tasks.

Cognitive bias	Mechanism	Source of bias in functional model (ACT-R)
Confirmation bias	Attentional effect (seeking)	Feature selection behavior such as selecting SIGINT too early. Blended retrieval during layer choice using stored utilities.
	Overscaling in rule application (weighing)	Bias in blended retrieval of mappings from likelihood factor to revised probability (low value). Weighted-distance utilities used for layer selections shows confirmation bias in weighing.

Anchoring in learning	Underscaling in rule application	Bias in blended retrieval of mappings from likelihood factor to revised probability (high values)
	Centroid computation	Inertia from centroid estimates to consolidated values to DM. Productions encoding thresholds in distance for centroid updating

Representativeness	Base-rate neglect	Base rate not a cue for matching to a category. Compute distance to category centroid rather than cloud of events. Blended retrievals ignore number of events

Probability matching	Resource allocation	Use of instance-based learning leads to tendency of risk aversion against winner-take-all instances, leading to the tendency for the blended retrieval of instances between pure probability matching and winner-take-all

**Table 5:** S1, S2, RSR (RMR for Task 6), and linear regression (*r* ^2^) scores broken down by task and layer.

Task	S1 score			S2 score			RSR/RMR
	Model	Human	*R* ^2^	Model	Human	*r* ^2^	
1	78.1	68.7	.929*	55.0	69.1	.219	.650
2	68.2	53.7	.313*	78.7	79.1	.990*	.799
3	82.6	74.5	.001	45.2	45.3	.253*	.595
4-1	92.2	75.6	.730*				.761
4-2	92.7	76.2	.461*	47.7	44.0	.510*	.906
5-1	96.6	68.1	.037				.856
5-2	91.5	77.4	.078				.776
5-3	85.3	69.8	.115				.780
5-4	82.3	66.3	.262*	40.4	45.2	.637*	.618
6	91.2	91.0	.867*	34.8	31.2	.902*	.788

*P < .01.

**Table 6:** Set of S1, S2, RSR (RMR in Task 6), and linear regression (*r* ^2^) scores broken down by task for novel dataset and participants.

Task	S1 score			S2 score			RSR/RMR
	Model	Human	*R* ^2^	Model	Human	*r* ^2^	
1	81.7	80.7	.011	59.1	63.4	.141	.625
2	68.5	78.9	.347*	54.2	54.6	.765*	.534
3	72.1	79.7	.121	34.7	73.8	.701*	.692
4	94.4	87.6	.006	47.5	46.7	.992*	.893
5	84.5	84.5	.000	42.0	45.5	.943*	.864
6	85.3	88.3	.447*	48.4	44.6	.990*	.854

*P < .01.

## Appendix

### PROBs Handbook

Figures 16, 17, 18, 19, and 20 were the individual pages of the PROBs (Probabilistic Decision Rules) handbook explaining how to update category likelihoods based on the information revealed by each feature.

## References

[1] Anderson JR (2007). *How Can the Human Mind Occur in the Physical Universe?*.

[2] Anderson JR, Bothell D, Byrne MD, Douglass S, Lebiere C, Qin Y (2004). An integrated theory of the mind. *Psychological Review*.

[3] Klein G, Moon B, Hoffman RR (2006). Making sense of sensemaking 1: alternative perspectives. *IEEE Intelligent Systems*.

[4] Klein G, Moon B, Hoffman RR (2006). Making sense of sensemaking 2: a macrocognitive model. *IEEE Intelligent Systems*.

[5] Pirolli P, Card SK The sensemaking process and leverage points for analyst technology.

[6] Russell DM, Stefik MJ, Pirolli P, Card SK The cost structure of sensemaking.

[7] Dervin B, Dervin B, Foreman-Wenet L, Lauterbach E (2003). From the mind’s eye of the user: the sense-making of qualitative-quantitative methodology. *Sense-Making Methodology Reader: Selected Writings of Brenda Dervin*.

[8] Weick K (1995). *Sensemaking in Oragnizations*.

[9] Heuer RJ (1999). *Psychology of Intelligence Analysis*.

[10] Hutchins SG, Pirolli P, Card SK, Hoffman RR (2007). What makes intelligence analysis difficult? A cognitive task analysis of intelligence analysts. *Expertise Out of Context*.

[11] Militello LG, Hutton RJB (1998). Applied cognitive task analysis (ACTA): a practitioner’s toolkit for understanding cognitive task demands. *Ergonomics*.

[12] Schraagen JM, Chipman SF, Shalin VL (2000). *Cognitive Task Analysis*.

[13] Posner MI, Goldsmith R, Welton KE (1967). Perceived distance and the classification of distorted patterns. *Journal of Experimental Psychology*.

[14] Lefebvre C, Dell’acqua R, Roelfsema PR, Jolicæur P (2011). Surfing the attentional waves during visual curve tracing: evidence from the sustained posterior contralateral negativity. *Psychophysiology*.

[15] Ashby FG, Maddox WT (1992). Complex decision rules in categorization: contrasting novice and experienced performance. *Journal of Experimental Psychology*.

[16] Stocco A, Lebiere C, O’Reilly RC, Anderson JR, Samsonovich AV, Jóhannsdóttir KR, Chella A, Goertzel B (2010). The role of the anterior prefrontal-basal ganglia circuit as a biological instruction interpreter. *Biologically Inspired Cognitive Architectures 2010*.

[17] Ryle G (1949). *The Concept of Mind*.

[18] Miller GA (1956). The magical number seven, plus or minus two: some limits on our capacity for processing information. *Psychological Review*.

[19] Simon HA (1974). How big is a chunk?. *Science*.

[20] Lebiere C (1999). The dynamics of cognition: an ACT-R model of cognitive arithmetic. *Kognitionswissenschaft*.

[21] Taatgen N, Lebiere C, Anderson JR, Sun R (2006). Modeling paradigms in ACT-R. *Cognition and Multi-Agent Interaction: From Cognitive Modeling to Social Simulation*.

[22] Card SK, Moran TP, Newell A (1983). *The Psychology of Human-Computer Interaction*.

[23] Newell A (1990). *Unified Theories of Cognition*.

[24] Anderson JR (1982). Acquisition of cognitive skill. *Psychological Review*.

[25] Anderson JR (1993). *Rules of the Mind*.

[26] Gonzalez C, Lerch JF, Lebiere C (2003). Instance-based learning in dynamic decision making. *Cognitive Science*.

[27] Lebiere C, Gonzalez C, Warwick W (2010). Metacognition and multiple strategies in a cognitive model of online control. *Journal of Artificial General Intelligence*.

[28] O’Reilly RC, Hazy TE, Herd SA, Chipman S The leabra cognitive architecture: how to play 20 principles with nature and win!. *Oxford Handbook of Cognitive Science*.

[29] Ziegler M, Howard M, Zaldivar A Simulation of anchoring bias in a spatial estimation task due to cholinergic neuromodulation.

[30] Sun Y, Wang H The parietal cortex in sensemaking: spatio-attentional aspects.

[31] Thomson R, Lebiere C Constraining Bayesian inference with cognitive architectures: an updated associative learning mechanism in ACT-R.

[32] Lebiere C, Anderson JR A connectionist implementation of the ACT-R production system.

[33] Jilk DJ, Lebiere C, O’Reilly RC, Anderson JR (2008). SAL: an explicitly pluralistic cognitive architecture. *Journal of Experimental and Theoretical Artificial Intelligence*.

[34] Marr D (1971). Simple memory: a theory for archicortex. *Philosophical Transactions of the Royal Society of London B*.

[35] Rumelhart DE, McClelland JL, PDP Research Group (1986). *Parallel Distributed Processing: Explorations in the Microstructure of Cognition, Vol. 1: Foundations*.

[36] Lebiere C, Anderson JR, Reder LM Error modeling in the ACT-R production system.

[37] Anderson JR (1990). *The Adaptive Character of Thought*.

[38] Klippel A, Tappe H, Habel C, Freksa C, Brauer W, Habel C, Wender KF (2003). Pictorial representations of routes: chunking route segments during comprehension. *Spatial Cognition III*.

[39] Kosslyn SM (1994). *Image and Brain: The Resolution of the Imagery Debate*.

[40] Gogel WC, da Silva JA (1987). A two-process theory of the response to size and distance. *Perception & Psychophysics*.

[41] Wiest WM, Bell B (1985). Stevens’s exponent for psychophysical scaling of perceived, remembered, and inferred distance. *Psychological Bulletin*.

[42] da Silva JA (1985). Scales for perceived egocentric distance in a large open field: comparison of three psychophysical methods. *The American Journal of Psychology*.

[43] Aznar-Casanova JA, Matsushima EH, Ribeiro-Filho NP, da Silva JA (2006). One-dimensional and multi-dimensional studies of the exocentric distance estimates in frontoparallel plane, virtual space, and outdoor open field. *The Spanish Journal of Psychology*.

[44] Levin CA, Haber RN (1993). Visual angle as a determinant of perceived interobject distance. *Perception & Psychophysics*.

[45] Thomson R *The role of object size on judgments of lateral separation [Ph.D. dissertation]*.

[46] Dehaene S, Cohen L (1999). Language and elementary arithmetic: dissociations between operations. *Brain and Language*.

[47] Lebiere C, Gonzalez C, Martin M Instance-based decision making model of repeated binary choice.

[48] Erev I, Ert E, Roth AE (2010). A choice prediction competition: choices from experience and from description. *Journal of Behavioral Decision Making*.

[49] Wallach D, Lebiere C, Jimenez L (2003). Conscious and unconscious knowledge: mapping to the symbolic and subsymbolic levels of a hybrid architecture. *Attention and Implicit Learning*.

[50] Lebiere C, Gonzalez C, Warwick W A comparative approach to understanding general intelligence: predicting cognitive performance in an open-ended dynamic task.

[51] Klayman J, Busemeyer J, Hastie R, Medin DL (1995). Varieties of confirmation bias. *Decision Making from a Cognitive Perspective*.

[52] Klayman J, Ha Y-W (1987). Confirmation, disconfirmation, and information in hypothesis testing. *Psychological Review*.

[53] Nickerson RS (1998). Confirmation bias: a ubiquitous phenomenon in many guises. *Review of General Psychology*.

[54] Tversky A, Kahneman D (1974). Judgment under uncertainty: heuristics and biases. *Science*.

[55] Wason P (1960). On the failure to eliminate hypotheses in a conceptual task. *The Quarterly Journal of Experimental Psychology*.

[56] Wickens CD, Hollands JG (2000). *Engineering Psychology and Human Performance*.

[57] Cheikes BA, Brown MJ, Lehner PE, Adelman L (2004). Confirmation bias in complex analyses.

[58] Convertino G, Billman D, Pirolli P, Massar JP, Shrager J (2006). Collaborative intelligence analysis with CACHE: bias reduction and information coverage.

[59] Tolcott MA, Marvin FF, Lehner PE (1989). Expert decisionmaking in evolving situations. *IEEE Transactions on Systems, Man and Cybernetics*.

[60] Grabo C, Goldman J (2004). *Anticipating Surprise*.

[61] Anderson JR, Lebiere C (1998). *The Atomic Components of Thought*.

[62] Burns K, Montgomery H, Lipshitz R, Brehmer B (2004). Mental models and normal errors. *How Professionals Make Decision*.

[63] MITRE Technical Report IARPA’s ICArUS program: phase 1 challenge problem design and test specification.

[64] MITRE Technical Report A computational basis for ICArUS challenge problem design.

[65] Kahneman D, Frederick S, Holyoak KJ, Morrison RG (2005). A model of heuristic judgment. *The Cambridge Handbook of Thinking and Reasoning*.

